# Pro-inflammatory cytokine polymorphisms and interactions with dietary alcohol and estrogen, risk factors for invasive breast cancer using a post genome-wide analysis for gene–gene and gene–lifestyle interaction

**DOI:** 10.1038/s41598-020-80197-1

**Published:** 2021-01-13

**Authors:** Su Yon Jung, Jeanette C. Papp, Eric M. Sobel, Matteo Pellegrini, Herbert Yu, Zuo-Feng Zhang

**Affiliations:** 1 Translational Sciences Section, Jonsson Comprehensive Cancer Center, School of Nursing, University of California, Los Angeles, 700 Tiverton Ave, 3-264 Factor Building, Los Angeles, CA 90095 USA; 2 Department of Human Genetics, David Geffen School of Medicine, University of California, Los Angeles, Los Angeles, CA 90095 USA; 3 Department of Computational Medicine, David Geffen School of Medicine, University of California, Los Angeles, Los Angeles, CA 90095 USA; 4 Department of Molecular, Cell and Developmental Biology, Life Sciences Division, University of California, Los Angeles, Los Angeles, CA 90095 USA; 5 Cancer Epidemiology Program, University of Hawaii Cancer Center, Honolulu, HI 96813 USA; 6 Department of Epidemiology, Fielding School of Public Health, University of California, Los Angeles, Los Angeles, CA 90095 USA; 7 Center for Human Nutrition, David Geffen School of Medicine, University of California, Los Angeles, Los Angeles, CA 90095 USA

**Keywords:** Cancer, Computational biology and bioinformatics, Genetics, Immunology, Molecular biology, Biomarkers, Medical research, Molecular medicine, Oncology, Risk factors

## Abstract

Molecular and genetic immune-related pathways connected to breast cancer and lifestyles in postmenopausal women are not fully characterized. In this study, we explored the role of pro-inflammatory cytokines such as C-reactive protein (CRP) and interleukin-6 (IL-6) in those pathways at the genome-wide level. With single-nucleotide polymorphisms (SNPs) in the biomarkers and lifestyles together, we further constructed risk profiles to improve predictability for breast cancer. Our earlier genome-wide association gene-environment interaction study used large cohort data from the Women’s Health Initiative Database for Genotypes and Phenotypes Study and identified 88 SNPs associated with CRP and IL-6. For this study, we added an additional 68 SNPs from previous GWA studies, and together with 48 selected lifestyles, evaluated for the association with breast cancer risk via a 2-stage multimodal random survival forest and generalized multifactor dimensionality reduction methods. Overall and in obesity strata (by body mass index, waist, waist-to-hip ratio, exercise, and dietary fat intake), we identified the most predictive genetic and lifestyle variables. Two SNPs (*SALL1* rs10521222 and *HLA-DQA1* rs9271608) and lifestyles, including alcohol intake, lifetime cumulative exposure to estrogen, and overall and visceral obesity, are the most common and strongest predictive markers for breast cancer across the analyses. The risk profile that combined those variables presented their synergistic effect on the increased breast cancer risk in a gene–lifestyle dose-dependent manner. Our study may contribute to improved predictability for breast cancer and suggest potential interventions for the women with the risk genotypes and lifestyles to reduce their breast cancer risk.

## Introduction

Chronic inflammation may play an important role in the pathogenesis of non-inflammatory diseases, such as breast cancer, from tumor initiation through progression^1,2^. Activation of innate immunity creates a tissue microenvironment high in reactive oxygen and nitrogen species, leading to potential DNA damage and alterations in nearby cells^3–5^. The inflammatory response also elevates the circulating levels of cancer-promoting inflammatory cytokines such as C-reactive protein (CRP) and interleukin-6 (IL-6)^2^. These key pro-inflammatory biomarkers reflect different molecular pathways in the immune cascade in acute and chronic immune responses but may be interrelated in carcinogenesis, yielding a congruent association with breast cancer risk. For example, IL-6, upregulated by macrophages and adipose tissue, promotes breast tumor initiation and progression^6,7^. CRP, a major acute-phase reactant and a biomarker of chronic low-grade inflammation, partially induced by IL-6, has been associated with increased risk of breast cancer^8,9^. The carcinogenetic mechanisms of these markers are partially understood. IL-6 regulates aromatase activity responsible for estrogen production in adipose tissue, which is important in developing postmenopausal breast cancer^10,11^. CRP levels are attenuated by prolonged inhibition of cyclooxygenase-2 action (promoting estrogen formation in adipose tissue)^11,12^. Thus, IL-6 and CRP may be involved in inflammatory pathways connected to breast cancer tumorigenesis.

Given the relationships between those inflammatory markers and breast cancer risk, genetic variants involved in the biomarkers’ functional and structural regulation may have potential implication in the causal pathway, affecting the risk of breast cancer. Previous genomic epidemiology studies for the associations between CRP/IL-6-related genome-wide genetic variants and breast cancer risk are limited and mostly showed null results^13–17^, while only a few reported a marginal effect on breast cancer risk^6^. The gene–phenotype pathway may not be connected to CRP and IL-6 alone, but also modulated by lifestyle pathways linked to obesity (overall and visceral)^15,18–25^, lipid metabolism^25,26^, high-fat diet, exercise, smoking, and alcohol^18,27–34^. Further, the inflammatory cytokines and the genetic markers have demonstrated different associations with breast cancer according to obesity^16,35^ and related lifestyle factors such as physical activity and dyslipidemia^36–38^. Thus, studying how those lifestyle factors modify and interact with gene and phenotype, leading to increased breast cancer susceptibility, may contribute to the understanding of the complex genotype–phenotype pathway and is important to develop a genetically targeted intervention tool for use in primary breast cancer prevention efforts.

In addition, immune-related etiologic pathways in breast cancer development may differ by menopausal status, probably due to the role of sex hormones in mediating the innate and adaptive immune systems. Our current study has focused on postmenopausal women who are vulnerable to a high incidence of inflammation^39^, obesity, and breast cancer (e.g., 80% of new cases occur in women age 50 years and older^40,41^). Using a large-scale postmenopausal women cohort from the Women’s Health Initiative Database for Genotypes and Phenotypes (WHI dbGaP) Study, we previously performed a genome-wide association (GWA) gene–environment (G × E) interaction study for CRP and IL-6 by addressing the pleiotropic effect of those biomarkers on the gene–phenotype relationship; we identified 88 top GWA single-nucleotide polymorphisms (SNPs)^42^. We have now extended the scope of modeled genetic factors by including 68 additional SNPs in relation to CRP and IL-6 from previous GWA studies that focused on European ancestry with independent replications^20,21,43,44^. We examined the association of those top GWA-based SNPs with primary invasive breast cancer risk overall and in obesity-related strata in which the SNPs were associated with CRP and IL-6 at genome-wide significance in our earlier GWA study^42^. This approach may allow us to elucidate an empirical pathway through which a substantial proportion of the susceptibility of GWA SNPs in CRP and IL-6 influences breast cancer risk through interactions with specific lifestyles (Figure S1).

In this study, we hoped to improve the predictability of breast cancer by better characterizing the genetic architecture of the inflammatory biomarkers that interact with lifestyle factors. We evaluated the GWA SNPs and 48 selected lifestyle factors together by conducting a two-stage multimodal random survival forest (RSF) analysis and ranked them according to their predictive value and accuracy for breast cancer. In addition, we applied a generalized multifactor dimensionality reduction (GMDR) model to characterize high-order gene–gene interactions and selected the best genetic prediction model^45–48^. Finally, with the most predictive SNPs and lifestyle factors selected via the RSF and GMDR, we constructed prediction models for breast cancer risk and estimated the combined and joint interaction effects of genotypes and lifestyles on the development of breast cancer. Ultimately, we tested the empirical hypothesis that the most-predictive genetic and lifestyle factors in combination increase the predictability of breast cancer risk in a synergistic manner.

## Material and methods

### Study population

Our study included healthy postmenopausal women enrolled in the WHI Harmonized and Imputed GWA Studies (GWASs) which was coordinated by dbGaP to contribute to a joint imputation and harmonization effort for GWASs within the 2 representative study arms, Clinical Trials and Observational Studies. The detailed study designs and rationale are described elsewhere^49,50^. Briefly, healthy women were enrolled in the WHI study between 1993 and 1998 at 40 clinical centers across the United States if they were 50–79 years old, postmenopausal, expected to stay near the clinical centers for at least 3 years after enrollment, and able to provide written informed consent. Participants were eligible for the WHI dbGaP study if they had met eligibility requirements for submission to dbGaP and provided DNA samples. The Harmonization and Imputation GWASs under the dbGaP study accession (phs000200.v12.p3) consist of 6 sub-studies (Table S1). Of the 16,088 women who reported their race or ethnicity as non-Hispanic white (Figure S2), in our earlier GWA GxE study, we applied the exclusion criteria (diabetes history; genetic data duplications; first- and second-degree relatives; and genetic quality control [QC] based on principal components), leaving 10,798 women. In the current study, we additionally excluded 619 with < 1 year follow-up period and/or a diagnosis of any type of cancer at enrollment, leaving a total of 10,179 women (94% of the eligible 10,798 GWA participants). These women had been followed up through August 29, 2014, with a mean of 16 years follow-up, and 537 of them had developed primary invasive breast cancer. The Institutional Review Boards of each WHI participating clinical center and the University of California, Los Angeles, approved this study. all methods were performed in accordance with the relevant guidelines and regulations.

### Data collection and breast cancer outcome

The coordinating clinical centers conducted data quality assurance periodically and collected participant information through self-administered questionnaires. In this study, we initially selected 48 variables measured at screening for our analysis on the basis of (1) their association with inflammation and breast cancer through the literature review^36,51–54^ and (2) preliminary analyses including univariate and stepwise multiple regression analyses and a multicollinearity test. Those variables include demographic and socioeconomic factors (age, education, marital status, family income, and employment); family histories of breast and colorectal cancers and diabetes; medical histories (depressive symptoms, hypertension, high cholesterol, and cardiovascular disease); lifestyles (cigarette smoking and exercise); dietary factors (dietary energy, alcohol intake, total sugar, fiber, fruit, and vegetable consumption; % calories from protein, carbohydrates, saturated fatty acids [SFA], monounsaturated FA [MFA], and polyunsaturated FA [PFA]); and reproductive histories (history of hysterectomy, removal of one or both ovaries, ages at menarche and menopause, pregnancy, breast feeding, oral contraceptive (OC) use, and use of exogenous estrogen [E] only and E plus progestin [E + P]). We also included anthropometric variables, including height, weight, and waist and hip circumferences, which had been measured by trained staff.

The breast cancer outcomes were determined via a centralized review of medical charts by a committee of physicians on the basis of pathology or cytology reports. The time from enrollment to breast cancer development, censoring, or study end point was calculated and represented in years. Cancer cases were coded using the National Cancer Institute’s Surveillance, Epidemiology, and End-Results guidelines^55^.

### Genotyping

We extracted genotyped data from the WHI dbGaP Harmonized and Imputed GWASs. Details of the data-cleaning process have been previously discussed^42,56^. Briefly, the genotypes were normalized to the reference panel GRCh37, and imputation was conducted via 1000 Genomes reference panels^57^. SNPs for harmonization were checked for pairwise concordance among all samples across the GWASs. The initial data QC included SNP filtering with a missing-call rate of < 2% and a Hardy–Weinberg equilibrium of p ≥ 1E–04. The second QC step included SNPs with ${\widehat{R}}^{2}\ge 0.6$ imputation quality^58^ but excluded individuals with a KING kinship estimate > 0.088^59^.

### Statistical analysis

Differences in participants’ baseline characteristics and allele frequencies by breast cancer development were examined with unpaired 2-sample *t* tests (for continuous variables) and chi-squared tests (for categorical variables). If continuous variables were skewed or had outliers, Wilcoxon’s rank-sum test was conducted. Our previous GWA analysis evaluated the gene–lifestyle interactions via stratifications defined by body mass index (BMI; cutoff, 30 kg/m^2^), waist circumstance (WST; cutoff, 88 cm), waist-to-hip ratio (WHR; cutoff, 0.85), metabolic equivalents (METs; cutoff, 10 h/week), and % calories from SFA (cut-off, 9%). The results (G × E formal test and stratified analysis) from the sub-GWASs were combined in a meta-analysis assuming a fixed-effect model. In this study, we performed an association study of the 88 SNPs identified in subgroups by obesity and obesity-related lifestyle variables with breast cancer risk in the identical subgroups. The additional 68 SNPs from other GWA studies were pulled together overall and in subgroups for the purpose of analysis.

In the current study, we conducted the RSF analysis. The RSF initially generates bootstrap samples using approximately 63% of the original data and grows a tree from each sample via a splitting rule to maximize survival differences across daughter nodes. This tree-building process is repeated numerous times (*n* = 5000 in this study), creating a forest of trees^60,61^. An ensemble cumulative hazard estimate was calculated from each tree and averaged over all trees for each individual and used to compute a predicted cumulative breast cancer incidence rate. Also, using this ensemble estimate and creating the out-of-bag (OOB) data (about 37% of the original data not used for bootstrapping), the OOB concordance index (c-index) was estimated, which is a measure of prediction performance conceptually similar to the area under the receiver operating characteristic (AUROC) curve^60,62^. The rank of each variable was determined on the basis of its predictability for breast cancer according to 2 predictive parameters: (1) minimal depth (MD), in which variables that have a small MD and split the tree close to the root are considered highly predictive and (2) variable importance (VIMP), computed as the difference between the OOB c-indexes from the original OOB data and from the permuted OOB data, in which variables that have greater VIMP values are the more predictive^63^. Because they use different prediction algorithms, we expect the variables’ ranking to differ to some degree. The RSF, a machine-learning and nonparametric tree-based ensemble method, accounts for nonlinearity and high-order interactions among variables, which may not be handled by a traditional regression method^63,64^. The RSF may thus provide a more accurate risk estimation.

We performed a 2-stage RSF analysis (Figure S3). In the first stage, we implemented an RSF on SNPs and lifestyle factors separately. Only those SNPs and lifestyle factors with distinctly low MD and high VIMP values were carried over in the second stage. In that second stage, we took a multimodal approach overall and in subgroups (by BMI, WHR, WST, MET, and SFA) by (1) comparing MD and VIMP measures in the plot, (2) computing the OOB c-index from the nested RSF model, and (3) estimating the incremental error rate of each variable in the nested sequence of RSF models from the top variable and calculating a dropping error rate. This RSF multimodal approach enabled us to exclude from the outset the SNPs and lifestyle factors that were not significantly associated with breast cancer, leading to increased statistical power and corrected type I error rate compared with the original RSF model^61^.

Further, we applied a GMDR model that is described in detail elsewhere^45–47^. The GMDR reduces high-dimensional multifactor prediction to a single dimension by the ratio of high vs. low risk, and thus detects the best gene–gene interaction model. It produces key predictability performance measures, including testing balance accuracy (TBA), cross-validation consistency (CVC), and sign p value. The model with the highest TBA, CVC 10/10, and p < 0.05 based on 1000-times permutation testing was considered the best model.

Multiple Cox proportional hazards regressions, with a test of proportional hazards via a Schoenfeld residual plot and ρ evaluation, were conducted to obtain hazard ratios (HRs) and 95% confidence intervals (CIs) for the single and combined effects of SNPs and lifestyle factors on breast cancer, with adjustment for covariates (Table 1). A 2-tailed p value < 0.05 was considered statistically significant, and multiple comparisons were adjusted by the Benjamini–Hochberg method^65^. GMDR v.1.0. and R v.3.5.2. (survival, survivalROC, randomForestSRC, ggRandomForests, gamlss, ggsurvplot, and forestplot packages) were used.

**Table 1:** Characteristics of participants, stratified by breast cancer.

Characteristic	Participants without breast cancer (n = 9642)		Participants with breast cancer (n = 537)	
	n	(%)	n	(%)
Age in years, mean (SD)	66	(6.65)	66	(6.64)
**Education**				
≤ High school	3476	(36.1)	164	(30.5)*
> High school	6166	(63.9)	373	(69.5)
**Family income**				
< $35,000	4344	(46.1)	207	(39.2)*
≥ $35,000	5088	(53.9)	321	(60.8)
**Family history of diabetes**				
No	6596	(71.1)	349	(66.9)*
Yes	2681	(28.9)	173	(33.1)
**Family history of breast cancer**				
No	7838	(81.3)	416	(77.5)*
Yes	1804	(18.7)	121	(22.5)
Depressive symptom^a^, mean (SD)	0.027	(0.097)	0.031	(0.115)*
Dietary alcohol per day in g, mean (SD)	6.01	(11.27)	8.50	(14.77)*
**Dietary alcohol per day**^**b**^				
< 18	8750	(90.7)	462	(86.0)*
≥ 18	892	(9.3)	75	(14.0)
% calories from protein, mean (SD)	16.66	(3.05)	16.85	(3.21)
% calories from SFA, median (range)	11.33	(2.22–32.39)	11.46	(3.73–21.50)
% calories from MFA, mean (SD)	12.70	(3.26)	12.75	(3.17)
% calories from PFA, mean (SD)	6.82	(2.08)	6.81	(2.09)
METs hour week^−1c^	11.04	(12.90)	10.28	(11.69)
**METs hour week**^**−1**c^				
≥ 10.0	4001	(41.5)	220	(41.0)
< 10.0	5641	(58.5)	317	(59.0)
**How many cigarettes per day**				
< 15	5432	(56.3)	250	(46.6)*
≥ 15	4210	(43.7)	287	(53.4)
BMI in kg/m^2^, mean (SD)	27.71	(5.32)	29.03	(5.68)*
**BMI**^**d**^				
< 30.0	6859	(71.1)	320	(59.6)*
≥ 30.0	2783	(28.9)	217	(40.4)
Waist circumference in cm, mean (SD)	86.57	(12.77)	90.0	(13.25)*
**Waist circumference**^d^				
≤ 88	5756	(59.7)	268	(49.9)*
> 88	3886	(40.3)	269	(50.1)
Hip circumference in cm, mean (SD)	106.3	(11.10)	109.4	(11.48)*
Waist-to-hip ratio, mean (SD)	0.813	(0.073)	0.822	(0.075)*
**Waist-to-hip ratio**^d^				
≤ 0.85	6895	(71.5)	356	(66.3)*
> 0.85	2747	(28.5)	181	(33.7)
Age at menarche in years, mean (SD)	13	(1.44)	12.5	(1.48)*
**Hysterectomy ever**				
No	6143	(63.7)	376	(70.0)*
Yes	3499	(36.3)	161	(30.0)
Age at menopause in years, mean (SD)	48	(6.23)	49	(5.82)*
Oral contraceptive duration in years, mean (SD)	5.7	(3.28)	5.2	(3.05)*
**Exogenous estrogen use (E**-**only) in years**				
Never	6697	(69.5)	411	(76.5)*
< 5	1361	(14.1)	51	(9.5)
5 to < 10	516	(5.4)	17	(3.2)
≥ 10	1068	(11.1)	58	(10.8)
**Exogenous estrogen use (E + P) in years**				
Never	7940	(82.3)	412	(76.7)*
< 5	927	(9.6)	64	(11.9)
5 to < 10	406	(4.2)	30	(5.6)
≥ 10	369	(3.8)	31	(5.8)

BMI, body mass index; E, estrogen; E + P, estrogen + progestin; SFA, saturated fatty acids; MET, metabolic equivalent; MFA, monounsaturated fatty acids; PFA, polyunsaturated fatty acids; RSF, random survival forest.

*p < 0.05, chi-squared or Wilcoxon’s rank-sum test.

^a^Depression scales were estimated using a short form of the Center for Epidemiologic Studies Depression Scale.

^b^Dietary alcohol per day was stratified at 18 g/day, where the cutoff level or higher fall within the high-risk group in the RSF model.

^c^Physical activity was estimated via recreational physical activity combining walking and mild, moderate, and strenuous physical activity. Each activity was assigned a MET value corresponding to intensity; the total MET hours week^−1^ was calculated by multiplying the MET level for the activity by the hours exercised per week and summing the values for all activities. The total MET was stratified into 2 groups, with 10 METs as the cutoff according to current American College of Sports Medicine and American Heart Association recommendations^102^.

^d^BMI, waist circumference, and waist-to-hip ratio were categorized at 30 kg/m^2^, 88 cm, and 0.85, respectively, where those cutoff levels or higher fall within the overall or visceral obese range (https://www.cdc.gov/obesity/adult/defining.html)^103^.

## Results

The allele frequencies of 156 GWA CRP/IL-6-related SNPs and baseline characteristics of participants are displayed in Tables S1 and 1, respectively. Breast cancer patients had relatively higher education, greater family income, and family history of diabetes and breast cancer, smoked more cigarettes/day, consumed more dietary alcohol/day, and were more depressed, obese both overall and viscerally, and taller. They also tended to experience early menarche and late menopause and had less history of hysterectomy and shorter duration of OC and E-only use, but longer duration of E + P use.

### Two-stage multimodal RSF and GMDR approach

With the 156 GWA SNPs and 48 lifestyle factors, we performed the two-stage RSF and GMDR (Figure S3) to determine the most predictive variables with the highest predictability and lowest prediction error for breast cancer risk. In the first stage, we estimated 2 predictability performance measures, MD and VIMP. For lifestyles and SNPs separately, we created a plot to compare those 2 measures and identified the strongest predictive lifestyle and genetic factors that were in agreement with high ranks (Figure S4) in overall analysis: 12 of 48 lifestyles and 13 of 156 SNPs. We further conducted the first stage of RSF for SNPs in the subgroups, which yielded the following results: 8 and 13 of 117 SNPs (BMI < 30 and ≥ 30, respectively); 14 and 7 of 70 SNPs (WHR ≤ 0.85 and > 0.85, respectively); 10 and 6 of 81 SNPs (WST ≤ 88 and > 88, respectively); 7 and 12 of 82 SNPs (METs ≥ 10 and < 10, respectively); and 19 and 12 of 116 SNPs (SFA < 9 and ≥ 9, respectively). All of the SNPs identified in this first stage of RSF were associated with CRP.

**Figure 1:**
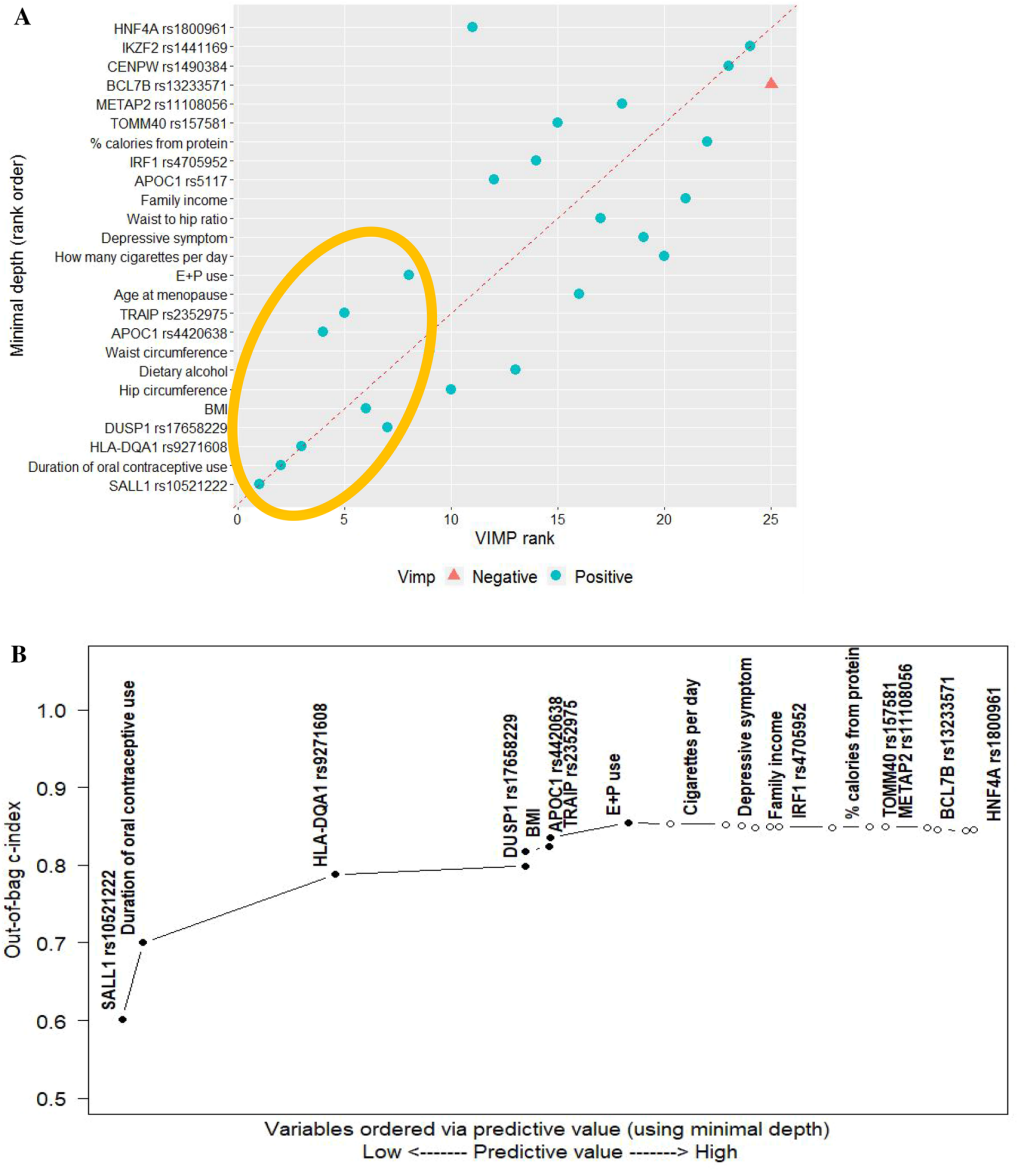
Overall analysis: the second stage of random survival forest (RSF) with 13 single-nucleotide polymorphisms and 12 behavioral factors selected from the first stage of RSF analysis. (**A**) Comparing minimal depth and VIMP rankings. (BMI, body mass index; E + P, exogenous estrogen + progestin; VIMP, variable of importance. 8 variables within the gold ellipse were identified as the most influential predictors). (**B**) Out-of-bag concordance index (c-index) (improvement in the out-of-bag c-index was observed when the top 8 variables [filled black circle] were added to the model, whereas other variables [open circle] did not further improve the accuracy of prediction)

Next, with the 12 lifestyles and selected SNPs together, overall and in subgroups, we conducted the second multimodal RSF to construct risk profiles with the most predictive variables. Particularly, in the overall group, we first computed the 2 measures MD and VIMP (Table 2) and compared them in a plot (Fig. 1A), in which a dashed red line represents agreement of the 2 measures. Both measures with high ranks indicated 5 SNPs (*SALL1* rs10521222; *HLA-DQA1* rs9271608; *DUSP1* rs17658229; *APOC1* rs4420638; and *TRAIP* rs2352975) and 3 lifestyles (duration of OC and E + P use and BMI) as the most influential variables for breast cancer. Second, we estimated the c-index (i.e., the AUROC) from the nested RSF model (Table 2) and plotted (Fig. 1B) where variables ranked by MD, identifying the same set of top variables (5 SNPs and 3 lifestyles). Those top variables substantially improved the c-index prediction accuracy, whereas others did not, suggesting that the c-index has complementary prediction ability. Last, we computed a dropping error rate for each variable in the nested sequence of RSF models (Table 2), and once again identified the same top 8 variables as the strongest contributors to reduce the error rate, thus improving the prediction accuracy. Further, using the GMDR method, we determined the best gene-by-gene interaction models up to 5 orders of interactions (Table 3), of which the one-factor model including *TRAIP* rs2352975 was the best predictive with the highest TBA of 0.5382 and CVC of 10/10 (p < 0.001).

**Table 2:** The second stage of random survival forest analysis: predictive value of variable for breast cancer in overall analysis.

Variable^a^	Minimal depth^b^	VIMP	C-index	Error^c^	Drop error^d^
*SALL1* rs10521222	1.9902	0.0554	0.6018	0.3982	0.1018
Duration of oral contraceptive use	2.0644	0.0541	0.7001	0.2999	0.0983
*HLA-DQA1* rs9271608	2.7866	0.0413	0.7878	0.2122	0.0878
*DUSP1* rs17658229	3.5022	0.0104	0.7990	0.2010	0.0111
BMI	3.5034	0.0113	0.8180	0.1820	0.0190
Hip circumference	3.5554	0.0073	0.8180	0.1820	− 1.00E−05
Dietary alcohol	3.5568	0.0049	0.8187	0.1813	7.00E−04
Waist circumference	3.5862	0.0075	0.8169	0.1831	− 0.0018
*APOC1* rs4420638	3.5908	0.0167	0.8242	0.1758	0.0073
*TRAIP* rs2352975	3.5954	0.0146	0.8359	0.1641	0.0118
Age at menopause	3.7836	0.0024	0.8392	0.1608	0.0033
Duration of E + P use	3.8872	0.0095	0.8539	0.1461	0.0147
How many cigarettes per day	4.0458	0.0015	0.8539	0.1461	− 9.00E−05
Depressive symptom	4.2520	0.0015	0.8525	0.1475	− 0.0014
Waist-to-hip ratio	4.3148	0.0022	0.8503	0.1497	− 0.0022
Family income	4.3646	0.0011	0.8479	0.1521	− 0.0024
*APOC1* rs5117	4.4182	0.0050	0.8494	0.1506	0.0016
*IRF1* rs4705952	4.4548	0.0046	0.8500	0.1500	0.0005
% calories from protein	4.6538	0.0007	0.8486	0.1514	− 0.0014
*TOMM40* rs157581	4.7930	0.0037	0.8499	0.1501	0.0013
*METAP2* rs11108056	4.8540	0.0017	0.8495	0.1505	− 0.0004
*BCL7B* rs13233571	5.0108	− 0.0001	0.8487	0.1513	− 0.0008
*CENPW* rs1490384	5.0488	0.0004	0.8458	0.1542	− 0.0029
*IKZF2* rs1441169	5.1550	0.0002	0.8439	0.1561	− 0.0019
*HNF4A* rs1800961	5.1846	0.0051	0.8455	0.1545	0.0016

BMI, body mass index; C-index, concordance index; E + P, exogenous estrogen + progestin; VIMP, variable of importance.

^a^Variables are ordered by minimal depth.

^b^Predictive value of variable was assessed via minimal depth in the nested random survival forest models. A lower value is likely to have a greater impact on prediction.

^c^The incremental error rate of each variable was estimated in the nested sequence of models starting with the top variable, followed by the model with the top 2 variables, then the model with the top 3 variables, and so on. For example, the 3rd error rate was estimated from the 3rd nested model (including the 1st, 2nd, and 3rd variables).

^d^The drop error rate was estimated by the difference between the error rates from the nested models with a prior and the corresponding variable. For example, the drop error rate of the 2nd variable was estimated by the difference between the error rates from the 1nd and 2rd nested models. The error rate for the null model is set at 0.5; thus, the drop error rate for the 1st variable was obtained by subtracting the error rate (0.3982) from 0.5.

**Table 3:** GMDR-based model for high-order gene–gene interactions in relation to breast cancer risk.

n	Model	TBA	*P* value	CVC
	**Overall**			
1	***TRAIP rs2352975***	**0.5382**	**0.0010**	**10/10**
2	*TRAIP* rs2352975, *SALL1* rs10521222	0.5270	0.0010	8/10
3	*TRAIP* rs2352975, *DUSP1* rs17658229, *SALL1* rs10521222	0.5271	0.0547	5/10
4	*TRAIP* rs2352975, *DUSP1* rs17658229, *SALL1* rs10521222, *APOC1* rs4420638	0.5249	0.0547	9/10
5	*TRAIP* rs2352975, *DUSP1* rs17658229, *SALL1* rs10521222, *APOC1* rs4420638, *HLA-DQA1* rs9271608	0.5215	0.0107	10/10
	**Overall non-obese group, BMI < 30 kg/m**^**2**^			
1	*APOC1* rs4420638	0.5179	0.1719	10/10
2	***APOC1 rs4420638, SALL1 rs10521222***	**0.5244**	**0.1712**	**10/10**
3	*APOC1* rs4420638, *SALL1* rs10521222, *HLA-DQA1* rs9271608	0.4980	0.6230	10/10
	**Overall obese group, BMI ≥ 30 kg/m**^**2**^			
1	***HNF1A-AS1 rs2243616***	**0.5551**	**0.0107**	**10/10**
2	*HNF1A-AS1* rs2243616, *SALL1* rs10521222	0.5546	0.0107	9/10
3	*HNF1A-AS1* rs2243616, *DUSP1* rs17658229, *SALL1* rs10521222	0.5533	0.0107	10/10
4	*HNF1A-AS1* rs2243616, *DUSP1* rs17658229, *SALL1* rs10521222, *HLA-DQA1* rs9271608	0.5510	0.0107	10/10
	Non-viscerally obese group, WHR ≤ 0.85*			
1	*APOC1* rs4420638	0.4924	0.8281	9/10
2	*DUSP1* rs17658229, *APOC1* rs4420638	0.5058	0.6230	9/10
3	*DUSP1* rs17658229, *APOC1* rs4420638, *SALL1* rs10521222	0.5009	0.6230	9/10
4	***DUSP1 rs17658229, APOC1 rs4420638, SALL1 rs10521222, HLA-DQA1 rs9271608***	**0.4851**	**0.9453**	**10/10**
	**Viscerally obese group, WHR > 0.85**			
1	*TRAIP* rs2352975	0.5306	0.1719	10/10
2	*TRAIP* rs2352975, *SALL1* rs10521222	0.5233	0.3770	5/10
3	***TRAIP rs2352975, SALL1 rs10521222, APOC1 rs4420638***	**0.5564**	**0.0547**	**10/10**
4	*TRAIP* rs2352975, *SALL1* rs10521222, *APOC1* rs4420638, *HLA-DQA1* rs9271608	0.5486	0.0547	10/10
	**Non-viscerally obese group, WST ≤ 88 cm**			
1	*APOC1* rs4420638	0.5190	0.1719	10/10
2	***APOC1 rs4420638, SALL1 rs10521222***	**0.5240**	**0.1719**	**10/10**
3	*APOC1* rs4420638, *SALL1* rs10521222, *HLA-DQA1* rs9271608	0.5047	0.3770	10/10
	**Viscerally obese group, WST > 88 cm**			
1	*TRAIP* rs2352975	0.5266	0.1719	10/10
2	*TRAIP* rs2352975, *SALL1* rs10521222	0.5307	0.1719	10/10
3	***TRAIP rs2352975, DUSP1 rs17658229, SALL1 rs10521222***	**0.5336**	**0.0547**	**10/10**
4	*TRAIP* rs2352975, *DUSP1* rs17658229, *SALL1* rs10521222, *HLA-DQA1* rs9271608	0.5291	0.1719	10/10
	Active group, MET ≥ 10.0*			
1	*HLA-DQA1* rs9271608	0.4872	0.9453	6/10
2	***HLA-DQA1 rs9271608, SALL1 rs10521222***	**0.4833**	**0.9893**	**10/10**
	**Inactive group, MET < 10.0**			
1	*SALL1* rs10521222	0.4727	0.9990	8/10
2	***SALL1 rs10521222, HLA-DQA1 rs9271608***	**0.4796**	**0.8281**	**10/10**
	**Low-fat diet group, % cal. from SFA < 9.0**			
1	*SALL1* rs10521222	0.4346	0.9990	6/10
2	*DUSP1* rs17658229, *HLA-DQA1* rs9271608	0.4305	1.0000	4/10
3	***DUSP1 rs17658229, HLA-DQA1 rs9271608, SALL1 rs10521222***	**0.4422**	**0.9990**	**10/10**
	**High-fat diet group, % cal. from SFA ≥ 9.0**			
1	*TRAIP* rs2352975	0.5463	0.0010	10/10
2	***TRAIP rs2352975, SALL1 rs10521222***	**0.5481**	**0.0010**	**10/10**
3	*TRAIP* rs2352975, *SALL1* rs10521222, *HLA-DQA1* rs9271608	0.5458	0.0010	10/10

BMI body mass index; CVC, cross-validation consistency; GMDR, generalized multifactor dimensionality reduction; MET, metabolic equivalent; SFA, saturated fatty acids; TBA, testing balance accuracy; WHR, waist-to-hip ratio; WST, waist circumference. Models in bold face are considered the best, with the highest TBA, 10/10 CVC, and p < 0.05.

*The models have either the highest TBA or 10/10 CVC, without statistical significance. By placing a greater importance on 10/10 CVC, the best model was selected.

For each of the obesity strata (BMI, WHR, WST, MET, and SFA), we continuously applied those multimodal (Tables S2.1–10 and Figures S5–9) and GMDR (Table 3) approaches, and determined the strongest predictive markers with the most common 6 SNPs (*TRAIP* rs2352975, *DUSP1* rs17658229, *HLA-DQA1* rs9271608, *SALL1* rs10521222, *HNF1A-AS1* rs2243616, and *APOC1* rs4420638) and 5 lifestyle factors (dietary alcohol intake, E + P and OC use, BMI, and hip circumference).

### Combined and joint effects of the most influential SNPs and lifestyles on breast cancer risk

**Figure 2:**
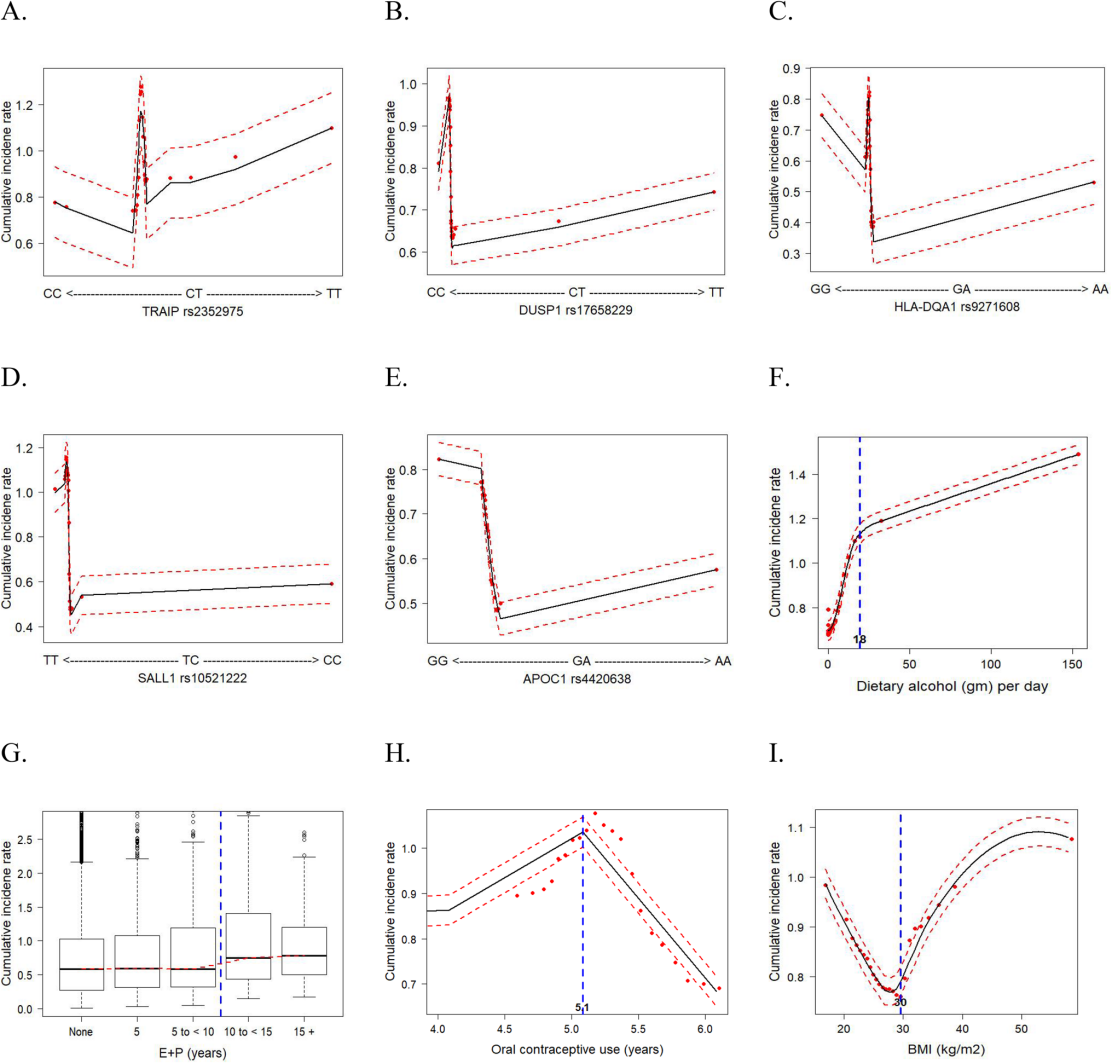
Cumulative breast cancer incidence rate for the 9 most influential variables (5 SNPs and 4 behavioral factors) based on random survival forest analyses. (E + P, exogenous estrogen + progestin; SNPs, single-nucleotide polymorphisms. Dashed red lines indicate 95% confidence intervals).

By accounting for confounding factors and the nonlinearity of each variable via the RSF method, we estimated the predicted cumulative incidence rate of breast cancer (Fig. 2). The genotypes of each SNP were originally continuous variables and then categorized accordingly for further analysis with the following risk genotypes (Fig. 2A–E): *TRAIP* rs2352975 CT + TT, *DUSP1* rs17658229 CC, *HLA-DQA1* rs9271608 GG, *SALL1* rs10521222 TT, and *APOC1* rs4420638 GG. Also, by using a cutoff value bisecting variables (Fig. 2F–I), high-risk lifestyle groups were defined as ≥ 18 g/day of alcohol consumption, ≥ 10 years of E + P use, < 5 years of past OC use, or ≥ 30 BMI and further analyzed as binary variables. With the best predictive GMDR-modeled SNPs and risk lifestyles overall and in subgroups, we developed multivariate models for breast cancer risk (Table S3). These results suggested a stronger individual effect of some SNPs than the rest of the SNPs and lifestyles on breast cancer risk, even after accounting for confounding factors.

The SNPs and lifestyles, when combined or jointly associated, displayed different patterns of breast cancer risk. In particular, in the overall non-obese (BMI < 30) group (Table 4), the best predictive SNPs and lifestyles were combined separately. When stratified by alcohol intake, high alcohol consumers (≥ 18 g/day) who had the maximum number of risk genotypes had a 4 times increased risk for breast cancer than low alcohol consumers (< 18 g/day) who had less or null-risk genotypes. Consistently, high alcohol consumers with one or more risk lifestyles had 3 times higher risk than low alcohol consumers with a null-risk lifestyle. When SNPs and lifestyles were combined, compared with the lowest-risk group (null risk for genotypes and lifestyles), the moderate-risk (high risk of either genotypes or lifestyles) and the highest-risk groups (high risk of both genotypes and lifestyles) had about 3 times and 6 times greater risk, respectively, suggesting a gene–lifestyle dose–response relationship. Further, when stratified by alcohol consumption, higher alcohol consumers with high risk of both genotypes and lifestyles had 10 times the excessive risk, compared with low alcohol consumers with low risk of both genotypes and lifestyles. This indicates a significant joint effect of alcohol intake with the SNPs and lifestyles on breast cancer risk in an additive model (G × E: HR = 1.15, p 0.547). Multiple testing was corrected to control the false-discovery rate. The analyses of the non-viscerally obese (WHR ≤ 0.85) group (Table 4) yielded similar results but with stronger combined and joint effects of risk genotypes and lifestyles with alcohol intake on breast cancer risk in both additive and multiplicative models (G × E: HR = 1.37, p 0.253).

**Table 4:** Stratification analysis by BMI and WHR: joint effect of dietary alcohol intake with combined risk genotypes and behavioral factors on breast cancer risk.

n	Total		n	Low dietary alcohol intake^a^		n	High dietary alcohol intake^a^		
	HR^b^ (95% CI)	p*		HR^b^ (95% CI)	p*		HR^b^ (95% CI)		p*
**Overall non-obese group, BMI < 30 kg/m**^**2**^** (n = 7179)**									
Risk genotypes (*SALL1* rs10521222 TT and *APOC1* rs4420638 GG)^c^									
0	Reference		2052	Reference		229	1.54 (0.72–3.29)		0.263
1	**2.38 (1.77–3.21)**	**1.21e−08**	4380	**2.38 (1.72–3.29)**	**1.75e−07**	518	**3.86 (2.53–5.88)**		**3.31e−10**
Behavioral factors (oral contraceptive use, E + P, and dietary alcohol intake)^d^									
0	Reference		4284	Reference		502	1.52 (1.00–2.31)		0.049
1	**1.80 (1.44–2.25)**	**2.73e−07**	2148	**1.72 (1.34–2.19)**	**1.56e−05**	245	**3.14 (2.06–4.78)**		**9.46e−08**
Risk genotypes combined with behavioral factors^e^									
0	Reference		1354	Reference		161	2.23 (0.83–6.04)		0.113
1	**3.17 (1.94–5.16)**	**3.59e−06**	3628	**3.22 (1.97–5.24)**	**2.83e−06**	409	**4.42 (2.40–8.15)**		**1.96e−06**
2	**5.51 (3.36–9.03)**	**1.30e−11**	1450	**5.13 (3.09–8.53)**	**2.69e−10**	177	**10.12 (5.46–18.78)**		**2.13e−13**
*p*_trend_		**1e−15**							
**Non-viscerally obese group, WHR ≤ 0.85 (n = 7251)**									
Risk genotypes (*DUSP1* rs17658229 CC, *HLA-DQA1* rs9271608 GG, *SALL1* rs10521222 TT, and *APOC1* rs4420638 GG)^c^									
0	Reference		4022	Reference		300	1.22 (0.65–2.28)		0.530
1	**2.75 (2.21–3.41)**	** < 2e−16**	2744	**2.59 (2.06–3.25)**	**4.22e−16**	185	**5.41 (3.55–8.23)**		**3.28e−15**
Behavioral factors (oral contraceptive use, E + P, hip circumference, and dietary alcohol intake)^d^									
0	Reference		2179	Reference		192	1.64 (0.81–3.33)		0.168
1	**1.63 (1.19–2.24)**	**0.002**	3027	**1.62 (1.17–2.23)**	**0.003**	204	**3.02 (1.78–5.13)**		**4.37e−05**
2	**2.68 (1.89–3.78)**	**2.43e−08**	1560	**2.50 (1.74–3.61)**	**8.08e−07**	89	**4.10 (2.11–7.99)**		**3.31e−05**
Risk genotypes combined with behavioral factors^e^									
0	Reference		1360	Reference		127	1.86 (0.54–6.35)		0.325
1	**3.26 (1.96–5.43)**	**5.68e−06**	3481	**3.33 (1.99–5.56)**	**4.26e−06**	238	**4.43 (2.16–9.05)**		**4.65e−05**
2	**7.05 (4.22–11.77)**	**8.29e−14**	1925	**6.60 (3.93–11.08)**	**8.82e−13**	120	**14.74 (7.71–28.19)**		**4.14e−16**
*p*_trend_		** < 2e−16**							

BMI, body mass index; CI, confidence interval; E + P, exogenous estrogen + progestin; HR, hazard ratio; WHR, waist-to-hip ratio. Numbers in bold face are statistically significant.

*p values were adjusted to correct for multiple testing via the Benjamini–Hochberg approach.

^a^In the overall non-obese subgroup, dietary alcohol was classified by a cutoff of 18 g/day (< 18 vs. ≥ 18); in the non-viscerally obese subgroup, dietary alcohol classified by a cutoff of 22 g/day (< 22 vs. ≥ 22);

^b^Multivariate regression for risk genotype analysis was adjusted by family income, BMI, waist and hip circumferences, depressive symptom, number of cigarettes per day, age at menopause, duration of oral contraceptive use, E + P use, % calories from protein, and dietary alcohol (in total analysis); for behavioral factor analysis, variables tested for stratification and joint effect were not included as covariates in the multivariate regression.

^c^The number of risk genotypes was defined as follows: [BMI < 30 subgroup] 0 (none or 1 risk allele) vs. 1 (2 risk alleles); [WHR ≤ 0.85 subgroup] 0 (none or 1/2/3 risk alleles) vs. 1 (4 risk alleles).

^d^The number of behavioral factors was defined as follows: [BMI < 30] 0 (null risk behavior) vs. 1 (1 or more risk behaviors); [WHR ≤ 0.85] 0 (null risk behavior) vs. 1 (1 risk behavior) vs. 2 (2 or more risk behaviors).

^e^The combined number of risk genotypes and behavioral factors was based on risk genotypes defined as 0 (low risk) and 1 (high risk) and based on behavioral factors defined as 0 (low risk) and 1 (high risk). The ultimate number of risk genotypes combined with behavioral factors was defined as 0 (low risk for genotypes and behaviors), 1 (high risk for either genotypes or behaviors), and 2 (high risk for both genotypes and behaviors).

**Figure 3:**
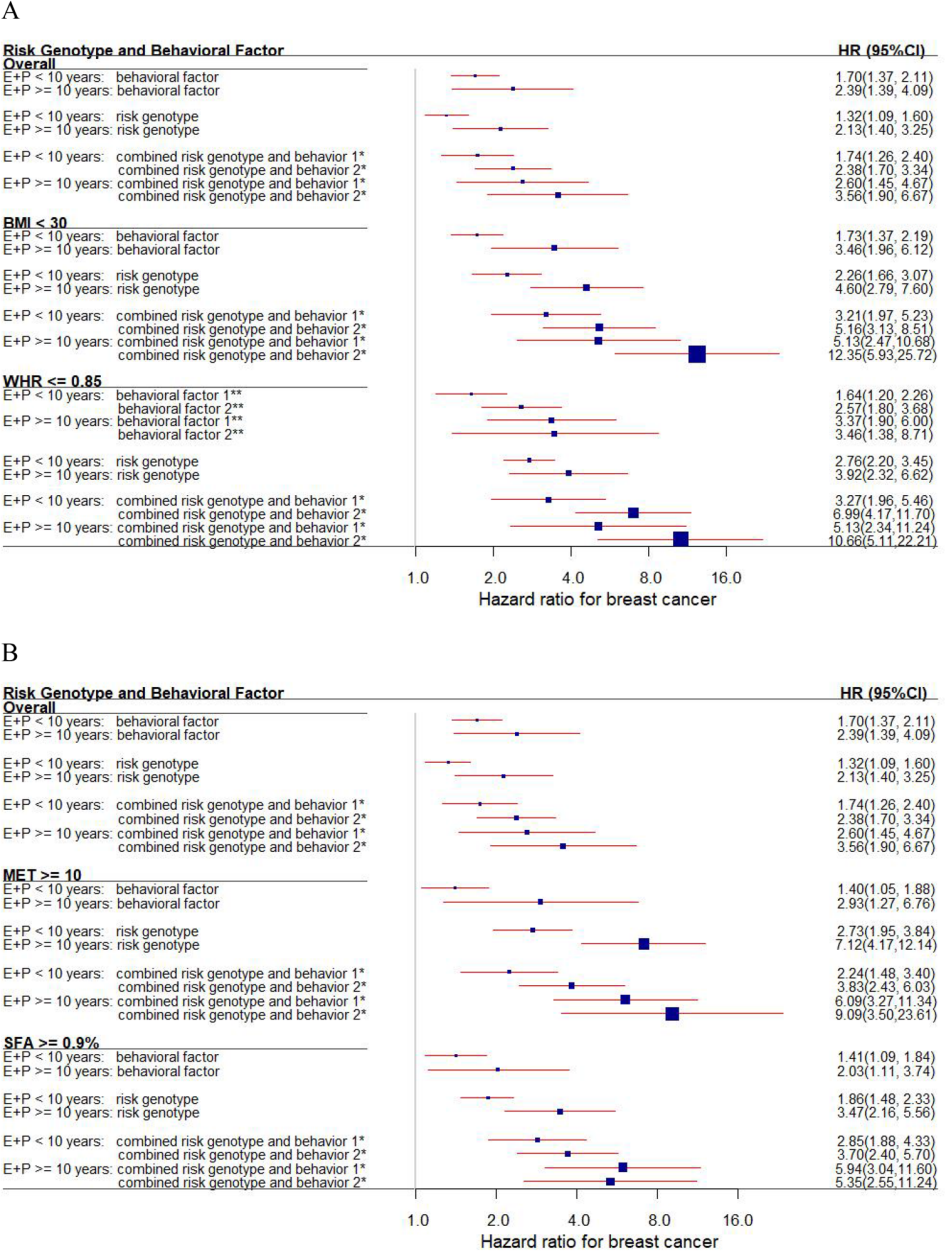
Forest plot of the joint effect of E + P use with risk behavioral factors and genotypes on breast cancer risk overall and in subgroups (**A** BMI < 30 and WHR ≤ 0.85; **B** MET ≥ 10 and SFA ≥ 9). The plot shows the independent and combined effect of risk behaviors and genotypes on breast cancer risk, jointly testing with E + P use, presented as the 95% CIs (indicated with red lines) and the estimates (proportional to the size of the blue squares). BMI, body mass index; CI, confidence interval; E + P, E + P, exogenous estrogen + progestin; HR, hazard ratio; MET, metabolic equivalent; SFA, saturated fatty acids; WHR, waist-to-hip ratio. * The combined number of risk genotypes and behavioral factors was based on risk genotypes defined as 0 (low risk: none or < total number of risk alleles) and 1 (high risk: combined all risk alleles) and based on behavioral factors defined as 0 (low risk: null risk behavior) and 1 (high risk: 1 or more risk behaviors). The ultimate number of risk genotypes combined with behavioral factors was defined as 0 (low risk for genotypes and behaviors), 1 (either high risk for genotypes or behaviors), and 2 (both high risk for genotypes and behaviors). ** The number of behavioral factors was defined as 0 (null risk behavior) vs. 1 (1 risk behavior) vs. 2 (2 or more risk behaviors).

We further evaluated the combined effect of SNPs and lifestyle factors and their joint effect with E + P use on breast cancer risk (Table S4) and determined that the risk genotypes and lifestyles, both separately and in combination, had a synergistic effect with longer use of E + P (≥ 10 years) on cancer risk. This pattern appeared more strongly in obesity strata (BMI, WHR, MET, and SFA) than in the overall group (Fig. 3).

## Discussion

An increasing number of population-based cancer genomic studies have incorporated environmental factors in the molecular causal pathway. Comprehending how lifestyle factors interact with genes and phenotypes, influencing risk for breast cancer, is important for constructing improved risk profiles, leading to the development of a gene–lifestyle combination intervention for primary cancer prevention efforts. Our 2-stage multimodal RSF and GMDR analyses identified the strongest predictive genetic and lifestyle variables overall and in obesity strata. The genetic effects in this study were associated with the SNPs involved in inflammatory cytokine pathways. The most common markers for breast cancer risk across the strata are 2 SNPs related to CRP (*SALL1* rs10521222 and *HLA-DQA1* rs9271608) and, consistent with previous studies^66–68^, 5 lifestyle factors such as alcohol intake, lifetime cumulative exposure to estrogen (post OC and E + P use), and overall and visceral obesity. The risk profiles that combined those influential variables presented a synergistic effect on the increased risk for breast cancer in a gene–lifestyle dose-dependent manner.

One SNP near *SALL1*, in relation to CRP, both overall and in the obesity strata, is associated with breast cancer risk. *SALL1* is a member of the *SALL* gene family, encoding a multiple zinc-finger transcription repressor that regulates organogenesis and development of embryonic stem cells^69–71^. The role of the *SALL* genes (particularly *SALL2* and *SALL4*) in tumorigenesis has recently been investigated as a tumor suppressor for ovarian and Wilms’ tumors^72,73^, hepatoblastoma, and gastric carcinoma^74,75^. However, the function of SALL1 in cancer development has not been determined. Few recent studies of in vivo RNAi screen and in vivo*/*in vitro breast cancer cells have implicated SALL1 as a tumor suppressor in breast cancer by inhibiting cancer cell growth, proliferation, and cell-cycle arrest, through the Nucleosome Remodeling and Deacetylase network^76^ or by regulating *CDH1*, a contributor to epithelial-to-mesenchymal transition^77^. Our finding of the *SALL1* SNP’s association with CRP at the GWA level and with breast cancer risk is supported by these previous biologic studies and further suggests the involvement of *SALL1* in immune mechanisms of breast cancer tumorigenesis.

*HLA-DQA1* belongs to the human leukocyte antigen (HLA) class II alpha chain paralogues, which increase immune system sensitivity by distinguishing its own proteins from foreign invaders^78,79^. HLA class II, the human version of the major histocompatibility complex (MHC) class II, regulates the antitumoral cellular immune response by presenting MHC antigen in tumor cells to the immune system, stimulating tumor infiltration of CD4 + T cells^80–82^. Several previous studies reported that the SNPs of HLA class II have implications in the carcinogenesis of specific cancers (e.g., ovarian^83^, squamous cell lung^84^, gastric^85^, and esophageal^86^ cancers), but limited studies in association with breast cancer have been conducted and were restricted to subjects other than Caucasians; further, the results were inconsistent^80,81^ or null^82^. Our study is the first to report the association of the *HLA-DQA1* SNP with breast cancer risk in non-Hispanic white women, suggesting that HLA class II plays a decisive role in the pathogenesis of breast cancer in this population by diminishing the efficacy of the antitumoral immune response. Also, this association would have been missed without the incorporation of obesity factors, which calls for further study of the biologic mechanism.

A number of epidemiologic studies have revealed that alcohol intake, even of a small amount (e.g., ≤ 1 drink [moderate]/day), can increase breast cancer risk in both pre- and post-menopausal women^66,87–90^. Notably, in postmenopausal women, few studies have examined the combined and joint effect of alcohol intake with other lifestyles^66–68^ or relevant genetic variants^91,92^ on breast cancer risk; in particular, the gene–lifestyle study results did not support a significantly increased risk among women who carried specific risk genotypes and had higher alcohol intake^91,92^. Molecular biologic mechanisms of alcohol-associated tumorigenesis in breast cancer may involve complicated pathways: an elevated level of estrogen by testosterone conversion; an increased level of insulin-like growth factors from the liver due to alcohol consumption^93,94^; and disruption of folate metabolism^95^. Also, acetaldehyde, derived from the metabolism of ethanol, is a carcinogenic metabolite that causes formation of DNA adducts and inhibits DNA repair and methylation patterns^90,96^. Further, high and regular alcohol intake may lead to a dietary deficiency of essential nutrients, making individuals susceptible to tumorigenesis^90^. Corresponding to this alcohol-response tumorigenic environment, and supported by previous research^66^, our study showed that more than moderate alcohol intake, jointly with the risk SNPs, substantially elevated the risk of breast cancer synergistically; and this synergistic effect occurred more strongly in the non-obese subgroups.

Another influential lifestyle factor in our study is the opposed E + P use that contributes to the lifetime cumulative exposure to estrogen. Synthetic progestin is a well-established risk factor for breast cancer^97–99^, with an affinity for androgen and mineralocorticoid receptors, leading to cell proliferation and anti-apoptosis^97,100^. Further, the joint effect of E + P use with the SNPs was profound in the non-obese subgroups, suggesting complementary pathways of sex hormones and obesity (i.e., the effect of sex hormones maximized in non-obese individuals with relatively lower hormone levels).

The amounts of daily dietary alcohol intake were obtained from self-reported food frequency questionnaires and then validated to be highly correlated with 1 month of food-diary records (*r* = 0.9)^101^. In addition, we confined our study population to non-Hispanic white postmenopausal women, limiting the generalizability of our study findings to other populations. Due to insufficient statistical power, we were unable to investigate the molecular subtypes of breast cancer. Despite several benefits from the 2-stage RSF multimodal and GMDR approaches, it can overfit the model owing to complicated analysis tasks, particularly in relatively small subgroups, so our results need to be replicated in an independent study with a large sample size.

Overall, in this study, the SNPs in proinflammatory cytokines previously identified as genome-wide significant had a synergistic effect on breast cancer risk by combining with lifestyle factors, including alcohol intake, lifetime cumulative exposure to estrogen, and obesity. Our findings warrant molecular biologic studies such as gene signature and aberrant cell signaling in relation to breast cancer in postmenopausal women who have a history of alcohol intake and estrogen use by different levels of obesity and related lifestyles. Our study may contribute to improved prediction accuracy and the ability to assess breast cancer risk, and suggest potential interventions for women who carry the risk genotypes, such as partial or absolute abstinence from alcohol intake, shorter duration of hormone therapy, and better weight control, potentially leading to an improved impact on the epigenetic aberrations and thus reducing the risk of breast cancer.

## Supplementary Information

Supplementary Information.

## Data Availability

The data that support the findings of this study are available in accordance with policies developed by the NHLBI and WHI in order to protect sensitive participant information and approved by the Fred Hutchinson Cancer Research Center, which currently serves as the IRB of record for the WHI. Data requests may be made by emailing helpdesk@WHI.org.
