# Supplementary material for: Pro-inflammatory cytokine polymorphisms and interactions with dietary alcohol and estrogen, risk factors for invasive breast cancer using a post genome-wide analysis for gene–gene and gene–lifestyle interaction
Source: Sci Rep. 2021 Jan 13;11:1058. doi: 10.1038/s41598-020-80197-1 (PMC7807068; doi:10.1038/s41598-020-80197-1)
Supplement: Supplementary file 1 — Supplementary Information. [file 41598_2020_80197_MOESM1_ESM.zip › Figure S1. Pathways_2020Mar17.docx]

Figure S1. Empirical pathways of pro-inflammatory SNPs, phenotypes, and breast cancer risk, modified by obesity status and obesity-lifestyle factors (note: pathways in red and blue lines were tested in our previous GWA G×E and current post-GWA analysis; yellow lines reflect hypothetical pathways). BMI, body mass index; CRP, C-reactive protein; IL-6, interleukin-6; SNP, single-nucleotide polymorphism; WHR, waist-to-hip ratio; WST, waist circumference.

**Phenotypes**

**(CRP/IL-6 levels)**

**X (CRP/IL-6 SNPs) Y (breast cancer risk)**

**Lifestyle factors:**

**obesity (BMI, WHR, and WST),**

**physical activity, and high-fat diet**
