# Supplementary material for: Pro-inflammatory cytokine polymorphisms and interactions with dietary alcohol and estrogen, risk factors for invasive breast cancer using a post genome-wide analysis for gene–gene and gene–lifestyle interaction
Source: Sci Rep. 2021 Jan 13;11:1058. doi: 10.1038/s41598-020-80197-1 (PMC7807068; doi:10.1038/s41598-020-80197-1)
Supplement: Supplementary file 1 — Supplementary Information. [file 41598_2020_80197_MOESM1_ESM.zip › Figure S2. Consort_BRCA_2020Nov30.docx]

Figure S2. Flow diagram of analytic cohort derived from the previous GWA G×E interaction study. (G×E, gene–environment; GWAS, genome-wide association study; *WHI GWASs participants were eligible if they had met eligibility requirements for submission to dbGaP and provided DNA samples within the 2 representative study arms, WHI Clinical Trials and Observational Studies.)

WHI Harmonized and Imputed GWASs*

Non-Hispanic white participants

(n = 16,088)

Previous GWAS G×E analysis for pro-inflammatory phenotypes

Excluded women (n = 2,714) who had diabetes at and/or after baseline

Applied exclusion criteria

13,374 women

Excluded women (n = 1,301) whose genetic information was duplicated in substudies (n = 1,210) and/or those with the first- and second-degree relatives (n = 91) in the dataset.

12,073 women

Excluded women (n = 1,275) whose genetic information did not satisfy the genetic quality assurance test (i.e., outliers based on Principal Components)

**10,179 women (94% of the 10,798; breast cancer = 537)**

**included in the post-GWA analysis with breast cancer**

Excluded women (n = 619) who had been followed up for less than 1 year and/or had been diagnosed with any cancer at enrollment

**10,798 women (90% of the eligible 12,073)**

**Included in previous GWAS G**×**E analysis**
