# Supplementary material for: Pro-inflammatory cytokine polymorphisms and interactions with dietary alcohol and estrogen, risk factors for invasive breast cancer using a post genome-wide analysis for gene–gene and gene–lifestyle interaction
Source: Sci Rep. 2021 Jan 13;11:1058. doi: 10.1038/s41598-020-80197-1 (PMC7807068; doi:10.1038/s41598-020-80197-1)
Supplement: Supplementary file 1 — Supplementary Information. [file 41598_2020_80197_MOESM1_ESM.zip › Figure S3_two-stage RSF.GMDR_2020July13.docx]

Figure S3. Two-stage RSF and GMDR

**< The First-Stage RSF >**

**Evaluation of the GWA top SNPs in relation to breast cancer risk in overall and obesity-specific subgroups**

12 lifestyle factors

13 SNPs

8 SNPs

13 SNPs

14 SNPs

7 SNPs

10 SNPs

6 SNPs

**GMDR**

7 SNPs

**Final (low fat):**

2 lifestyles; 3 SNPs

**Final (high fat):**

3 lifestyles; 2 SNPs

12 SNPs

**GMDR**

19 SNPs

12 SNPs

**< The Second-Stage Multimodal RSF and GMDR >**

1. **RSF (MD and VIMP)**
2. **OOB c-index (similar to the AUROC)**
3. **Incremental error rate of each variable in the nested sequence of RSF models**
4. **GMDR for G** × **G interation**

**Final (active):**

2 lifestyles; 2 SNPs

**Final (inactive):**

4 lifestyles; 2 SNPs

**Final (non-obese, WST):**

2 lifestyles; 2 SNPs

**Final (obese, WST):**

2 lifestyles; 3 SNPs

**Final (non-obese, WHR):**

4 lifestyle; 4 SNPs

**Final (obese, WHR):**

3 lifestyles; 3 SNPs

**GMDR**

**GMDR**

**GMDR**

**GMDR**

**Final (non-obese, BMI):**

3 lifestyles; 2 SNPs

**Final (obese, BMI):**

1 lifestyle; 1 SNP

**Final:** 3 lifestyles; 1 SNP
