# Supplementary material for: Pro-inflammatory cytokine polymorphisms and interactions with dietary alcohol and estrogen, risk factors for invasive breast cancer using a post genome-wide analysis for gene–gene and gene–lifestyle interaction
Source: Sci Rep. 2021 Jan 13;11:1058. doi: 10.1038/s41598-020-80197-1 (PMC7807068; doi:10.1038/s41598-020-80197-1)

Figure S4. The first stage of the random survival forest analysis, comparing minimal depth and VIMP rankings

A. Behavioral factors (BMI, body mass index; CVD, cardiovascular disease; E-only, exogenous estrogen; E+P, E + progestin; MFA, monounsaturated fatty acids; PFA, polyunsaturated fatty acids; SFA, saturated fatty acids; VIMP, variable of importance. Note: The 12 variables within the gold ellipse were identified as the most influential predictors)


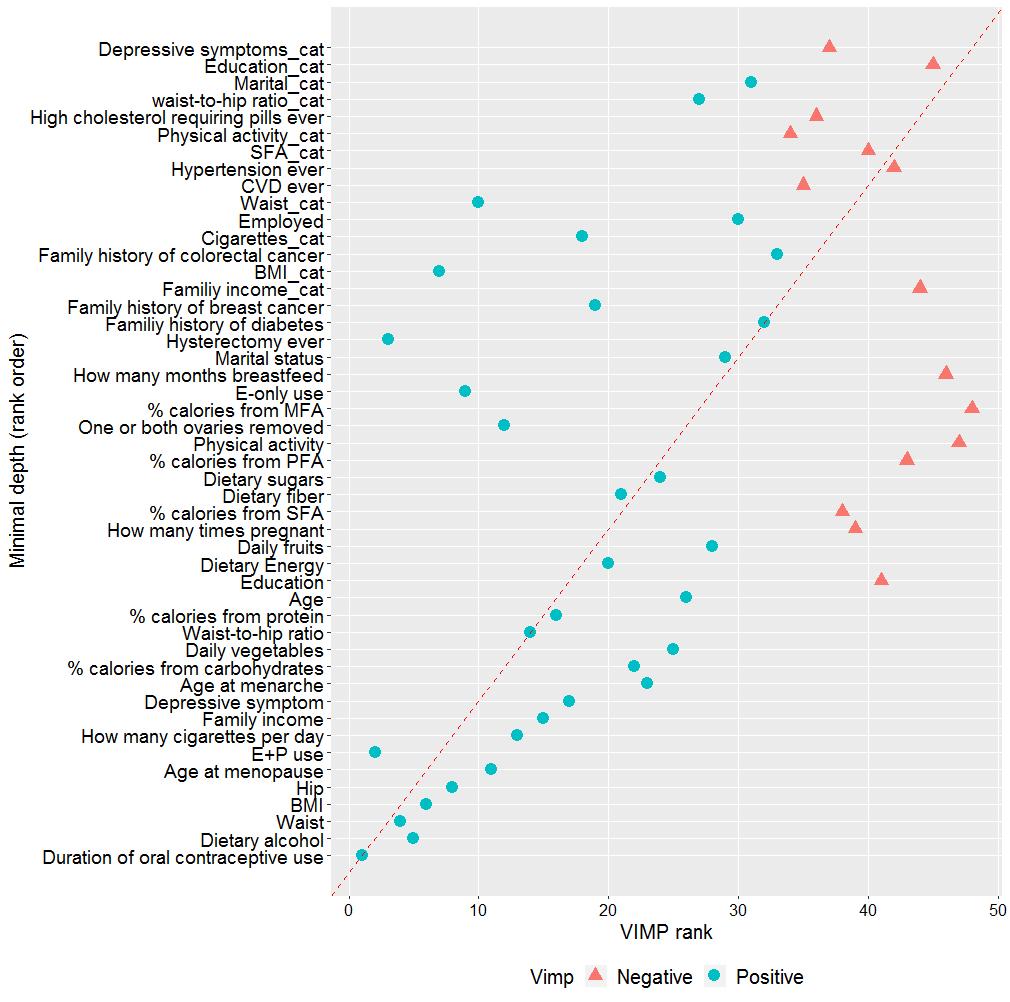


B. 156 SNPs in overall analysis (SNPs, single-nucleotide polymorphisms. Note: The 13 SNPs within the gold ellipse were identified as the most influential predictors)


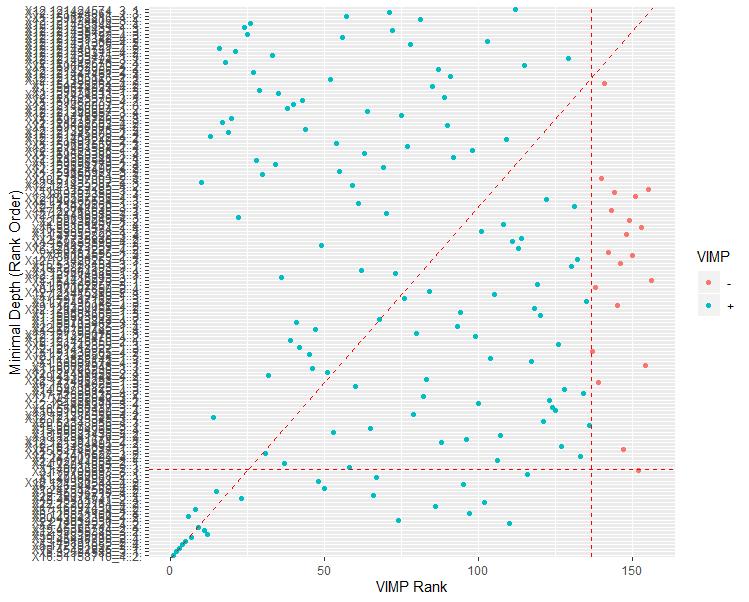


C. 117 SNPs in BMI-stratified analysis – overall non-obese group (BMI < 30) (BMI, body mass index. Note: The 8 SNPs within the gold ellipse were identified as the most influential predictors)


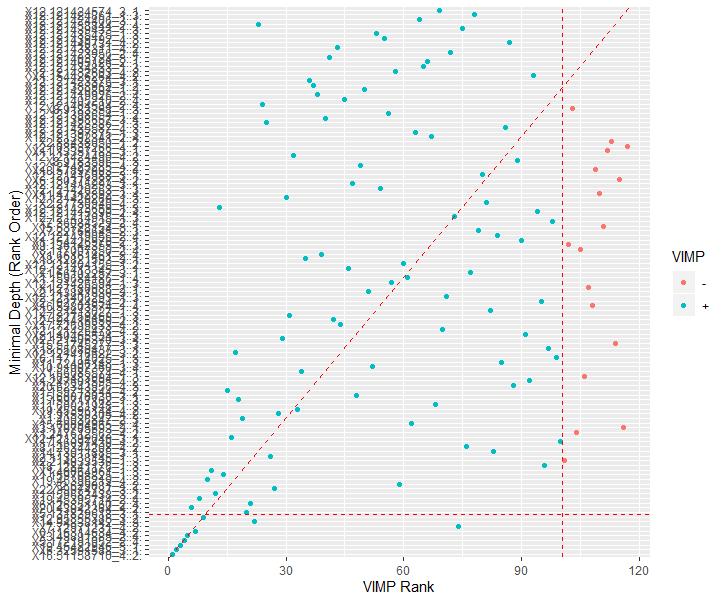


D. 117 SNPs in BMI-stratified analysis – overall obese group (BMI ≥ 30) (Note: The 13 SNPs within the gold ellipse were identified as the most influential predictors)


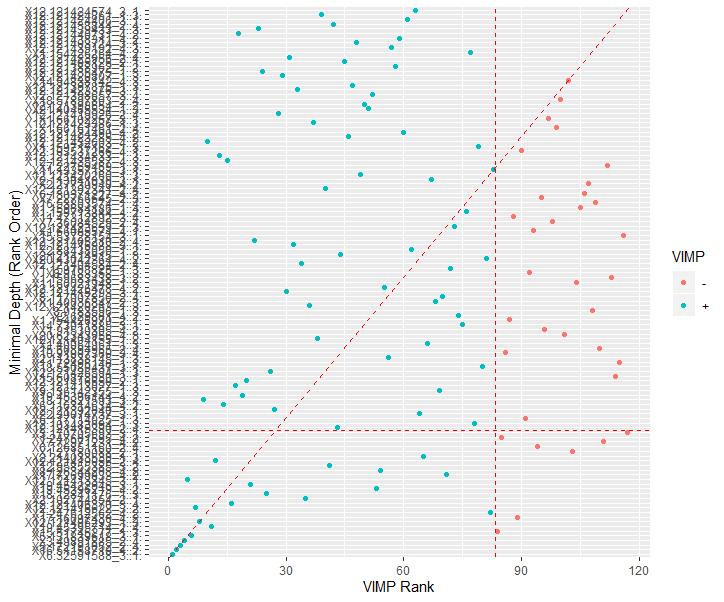


E. 70 SNPs in WHR-stratified analysis – non-viscerally obese group (WHR ≤ 0.85) (WHR, waist-to-hip ratio. Note: The 14 SNPs within the gold ellipse were identified as the most influential predictors)


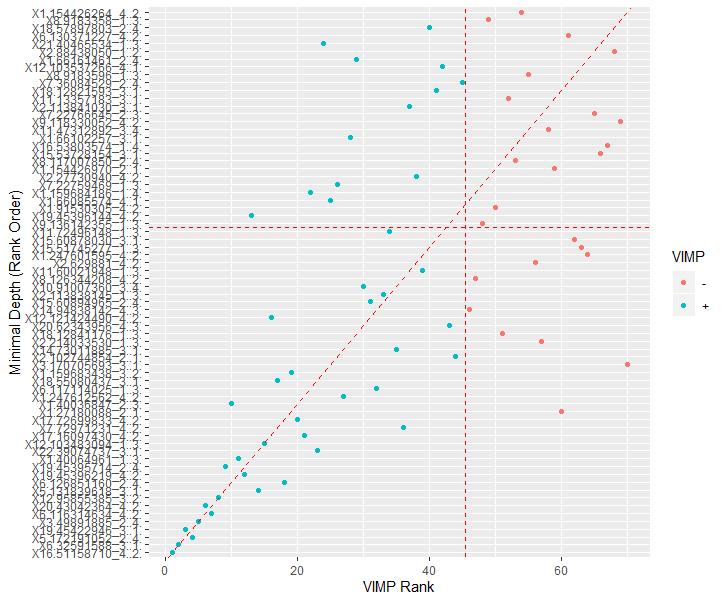


F. 70 SNPs in WHR-stratified analysis – viscerally obese group (WHR > 0.85) (Note: The 7 SNPs within the gold ellipse were identified as the most influential predictors)


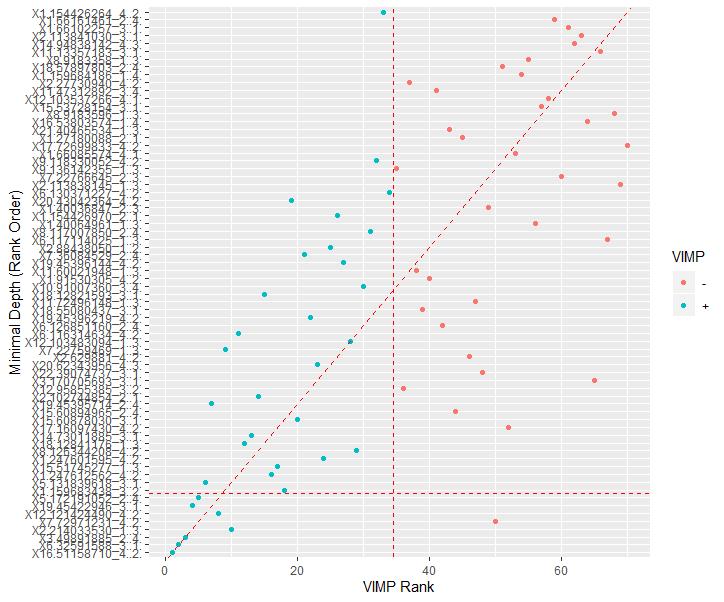


G. 81 SNPs in WST-stratified analysis – non-viscerally obese group (WST ≤ 88) (WST, waist circumference. Note: The 10 SNPs within the gold ellipse were identified as the most influential predictors)


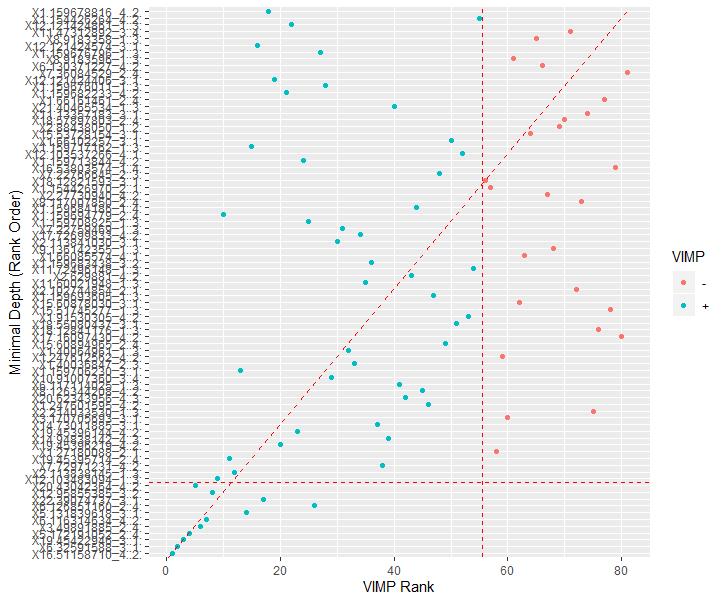


H. 81 SNPs in WST-stratified analysis – viscerally obese group (WST > 88) (Note: The 6 SNPs within the gold ellipse were identified as the most influential predictors)


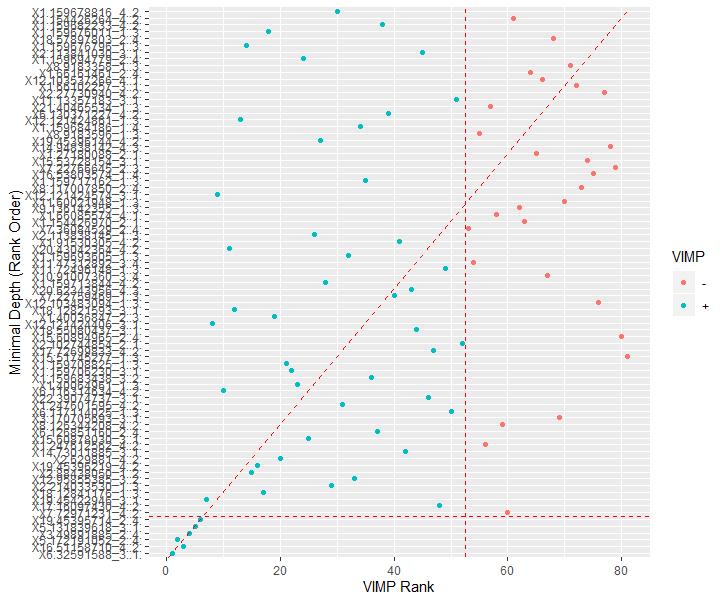


I. 82 SNPs in physical activity-stratified analysis - active group (MET ≥ 10) (MET, metabolic equivalent. Note: The 7 SNPs within the gold ellipse were identified as the most influential predictors)


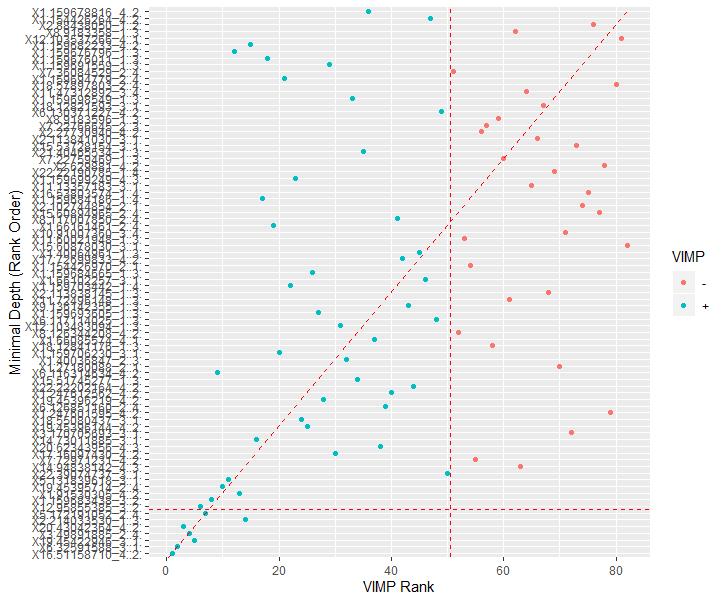


J. 82 SNPs in physical activity-stratified analysis - inactive group (MET < 10) (Note: The 12 SNPs within the gold ellipse were identified as the most influential predictors)


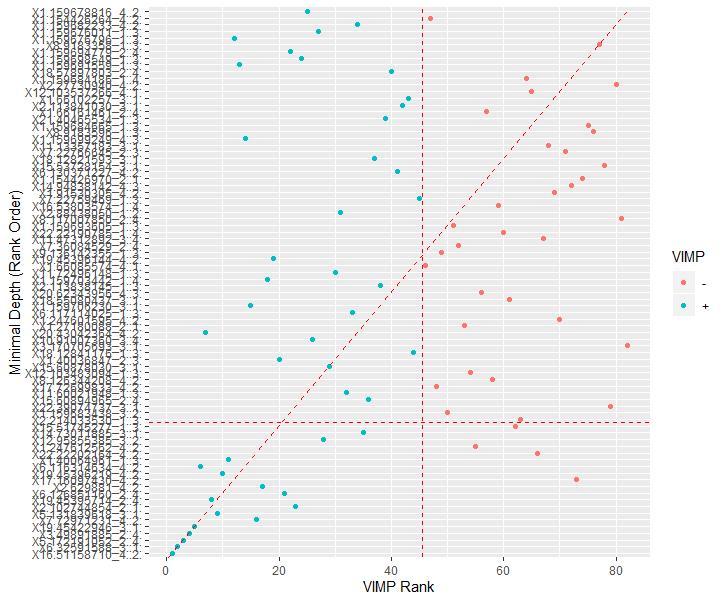


K. 116 SNPs in SFA-stratified analysis - % calories from SFA < 9.0 (SFA, saturated fatty acids. Note: The 19 SNPs within the gold ellipse were identified as the most influential predictors)


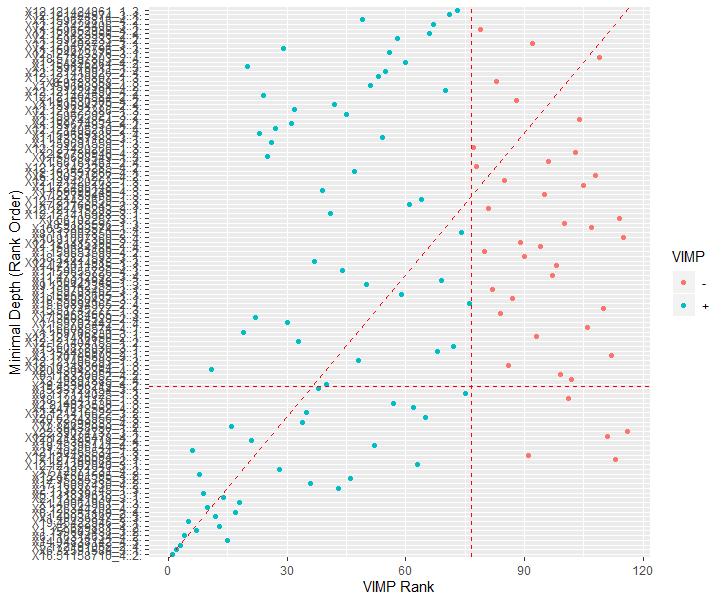


L. 116 SNPs in SFA-stratified analysis - % calories from SFA ≥ 9.0 (Note: The 12 SNPs within the gold ellipse were identified as the most influential predictors)


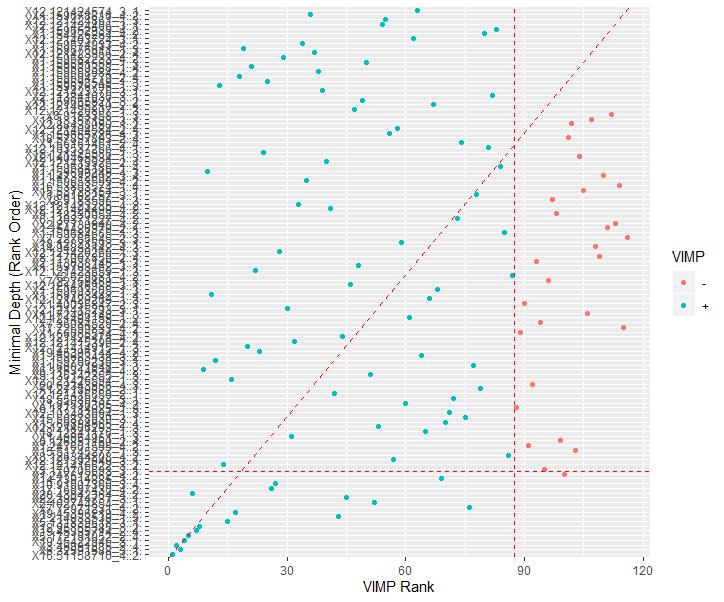

Supplement: Supplementary file 1 — Supplementary Information. [file 41598_2020_80197_MOESM1_ESM.zip › Figure S4_first stage_2020July13.docx]
