# Supplementary material for: Pro-inflammatory cytokine polymorphisms and interactions with dietary alcohol and estrogen, risk factors for invasive breast cancer using a post genome-wide analysis for gene–gene and gene–lifestyle interaction
Source: Sci Rep. 2021 Jan 13;11:1058. doi: 10.1038/s41598-020-80197-1 (PMC7807068; doi:10.1038/s41598-020-80197-1)

Figure S7. WST-stratified analysis: the second stage of the random survival forest (RSF) with 10 single-nucleotide polymorphisms (SNPs) and 12 behavioral factors selected from the first stage of the RSF in **non-viscerally obese group (WST ≤ 88, A1.A2.);** and with 6 SNPs and 12 behavioral factors in **viscerally obese group (WST > 88, B1.B2.)**. Variables within the gold ellipses were identified as the most influential predictors.

A1. Comparing minimal depth and VIMP rankings. (BMI, body mass index E+P, exogenous estrogen + progestin; VIMP, variable of importance)


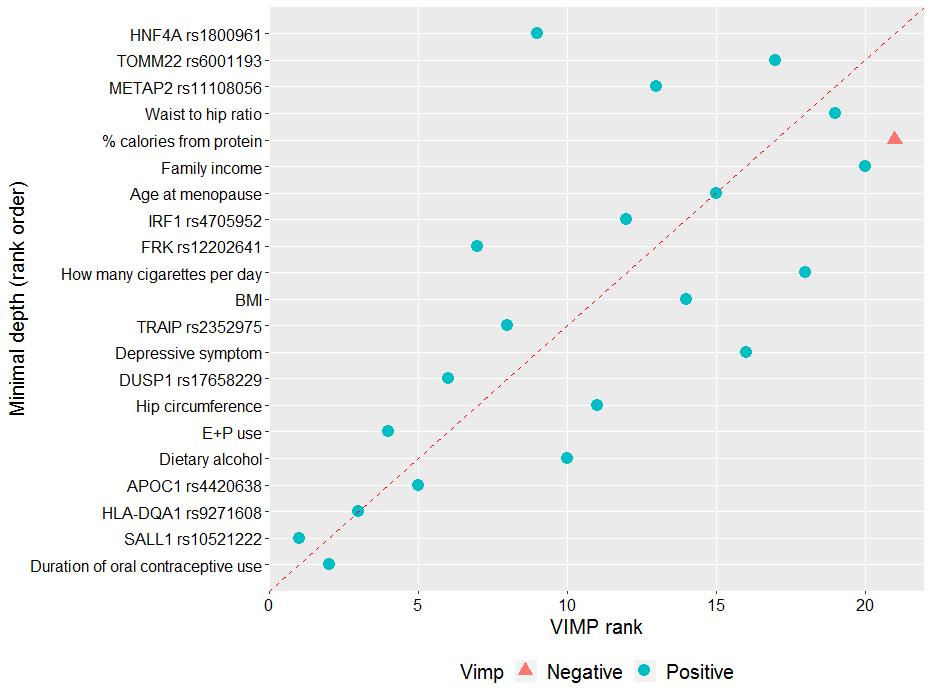


A2. Out-of-bag concordance index (c-index). (Improvement in the out-of-bag c-index was observed when the top 5 variables [●] were added to the model, whereas other variables [○] did not further improve the accuracy of prediction.)


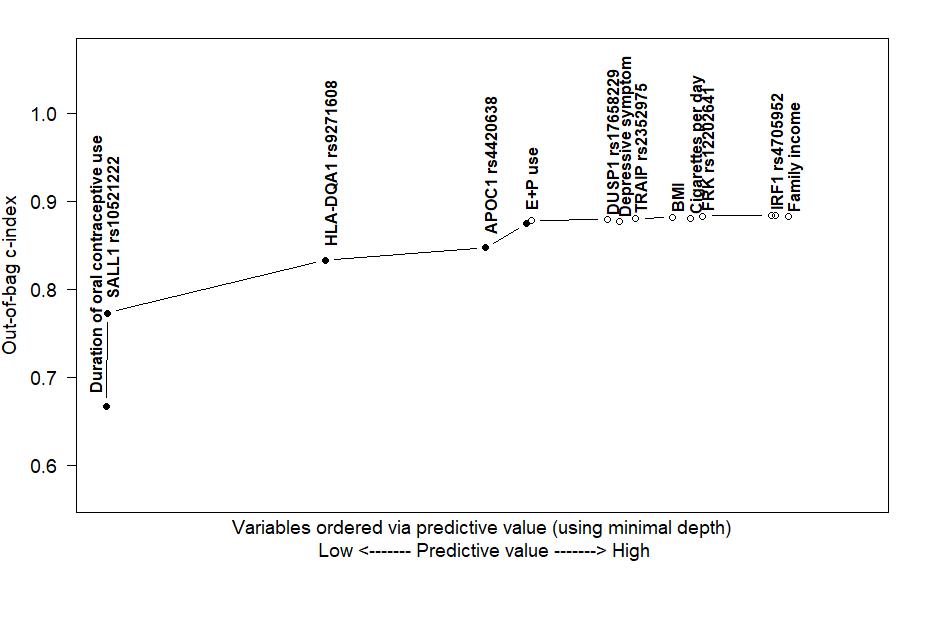


B1. Comparing minimal depth and VIMP rankings.


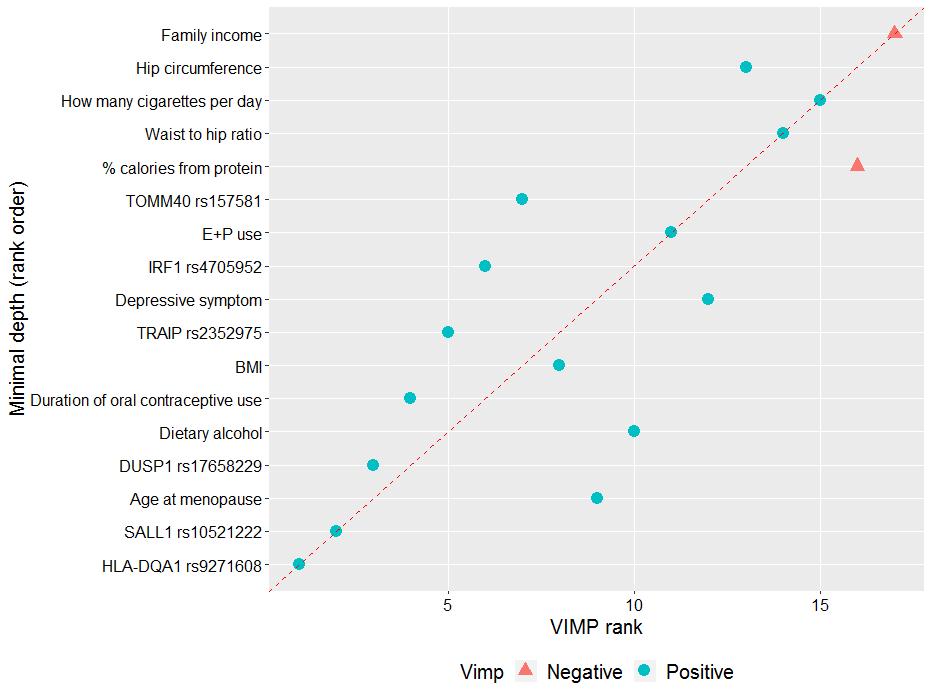
B2. Out-of-bag c-index. (Improvement in the out-of-bag c-index was observed when the top 4 variables [●] were added to the model, whereas other variables [○] did not further improve the accuracy of prediction.)


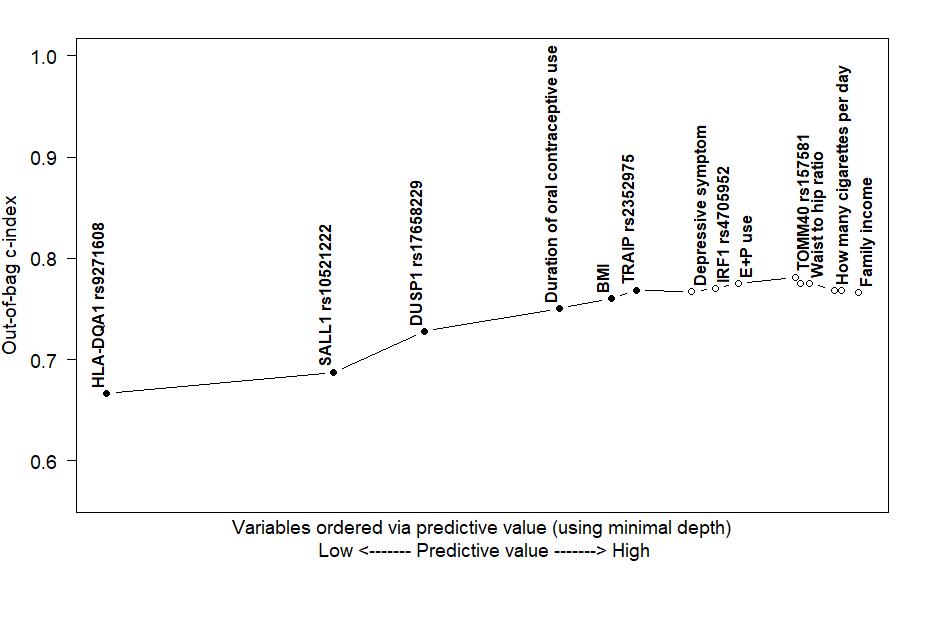

Supplement: Supplementary file 1 — Supplementary Information. [file 41598_2020_80197_MOESM1_ESM.zip › Figure S7_second stage file_WST_2020Mar17.docx]
