# Supplementary material for: Pro-inflammatory cytokine polymorphisms and interactions with dietary alcohol and estrogen, risk factors for invasive breast cancer using a post genome-wide analysis for gene–gene and gene–lifestyle interaction
Source: Sci Rep. 2021 Jan 13;11:1058. doi: 10.1038/s41598-020-80197-1 (PMC7807068; doi:10.1038/s41598-020-80197-1)
Supplement: Supplementary file 1 — Supplementary Information. [file 41598_2020_80197_MOESM1_ESM.zip › Table S1.SNP allele freq_2020Mar17.docx]

Table S1.1. Allele frequencies of 88 SNPs from our GWA analysis associated with pro-inflammatory phenotypes (total n = 10,798)

| **Chr** | **Position¥** | **Gene** | **SNP** | **Allele** | |  | **Alt Allele Frequency** | | | | | |
| --- | --- | --- | --- | --- | --- | --- | --- | --- | --- | --- | --- | --- |
|  |  |  |  |  |  |  | **AS264** | **GARNET** | **GECCOCYTO** | **GECCOINIT** | **HIPFX** | **WHIMS** |
|  |  |  |  | **Ref** | **Alt** |  | **n=1,603** | **n=2,382** | **n=1,177** | **n=216** | **n=1,909** | **n=3,511** |
| 1 | 159652939 | CRPP1 | rs2592887 | C | T |  | 0.41 | 0.41 | 0.41 | 0.38 | 0.40 | 0.39 |
| 1 | 159653599 | CRPP1 | rs1470515 | C | T |  | 0.40 | 0.39 | 0.40 | 0.37 | 0.38 | 0.38 |
| 1 | 159655726 | CRPP1 | rs2592902 | G | T |  | 0.40 | 0.39 | 0.40 | 0.37 | 0.38 | 0.38 |
| 1 | 159665921 | CRPP1 | rs2808624 | C | G |  | 0.40 | 0.39 | 0.40 | 0.36 | 0.38 | 0.38 |
| 1 | 159668984 | CRPP1 | rs11265257 | C | T |  | 0.40 | 0.39 | 0.40 | 0.36 | 0.38 | 0.38 |
| 1 | 159674933 | CRPP1 | rs876537 | C | T |  | 0.40 | 0.39 | 0.40 | 0.36 | 0.38 | 0.38 |
| 1 | 159676011 | CRPP1 | rs2808628 | G | A |  | 0.34 | 0.34 | 0.34 | 0.32 | 0.33 | 0.33 |
| 1 | 159676796 | CRPP1 | rs2808629 | G | A |  | 0.34 | 0.34 | 0.35 | 0.32 | 0.33 | 0.33 |
| 1 | 159678816 | CRPP1/CRP | rs2794520 | C | T |  | 0.34 | 0.34 | 0.34 | 0.32 | 0.33 | 0.33 |
| 1 | 159682233 | CRP | rs1205 | C | T |  | 0.34 | 0.34 | 0.34 | 0.32 | 0.33 | 0.33 |
| 1 | 159684665 | CRP | rs3091244 | G | A |  | 0.32 | 0.36 | 0.32 | 0.34 | 0.34 | 0.37 |
| 1 | 159689388 | CRP | rs2027471 | T | A |  | 0.35 | 0.35 | 0.35 | 0.33 | 0.33 | 0.33 |
| 1 | 159691559 | CRP | rs1341665 | G | A |  | 0.35 | 0.35 | 0.35 | 0.33 | 0.33 | 0.33 |
| 1 | 159693605 | CRP | rs2211320 | G | A |  | 0.33 | 0.33 | 0.33 | 0.31 | 0.31 | 0.32 |
| 1 | 159694779 | CRP | rs7551731 | T | C |  | 0.34 | 0.34 | 0.34 | 0.32 | 0.32 | 0.33 |
| 1 | 159698549 | CRP | rs7553007 | G | A |  | 0.34 | 0.34 | 0.34 | 0.31 | 0.32 | 0.32 |
| 1 | 159699249 | CRP | rs4546916 | G | T |  | 0.34 | 0.34 | 0.34 | 0.31 | 0.32 | 0.32 |
| 1 | 159703442 | CRP | rs4287174 | T | A |  | 0.34 | 0.34 | 0.34 | 0.31 | 0.32 | 0.32 |
| 1 | 159703462 | CRP | rs4428887 | A | G |  | 0.35 | 0.34 | 0.35 | 0.32 | 0.33 | 0.33 |
| 1 | 159706230 | CRP | rs12037186 | A | G |  | 0.33 | 0.33 | 0.33 | 0.30 | 0.31 | 0.32 |
| 1 | 159708825 | CRP/RP11-419N10.5 | rs12042360 | G | A |  | 0.16 | 0.17 | 0.18 | 0.16 | 0.16 | 0.15 |
| 1 | 159713844 | CRP/RP11-419N10.5 | rs12049404 | C | T |  | 0.16 | 0.17 | 0.18 | 0.16 | 0.16 | 0.15 |
| 1 | 159717162 | CRP/RP11-419N10.5 | rs11588887 | G | A |  | 0.16 | 0.17 | 0.18 | 0.16 | 0.16 | 0.15 |
| 9 | 118330052 | DEC1 | rs149109490 | T | C |  | 0.99 | 0.99 | 0.99 | 0.99 | 0.99 | 0.99 |
| 12 | 121380544 | HNF1A-AS1 | rs2649999 | T | C |  | NA | 0.66 | 0.67 | NA | 0.68 | 0.65 |
| 12 | 121384495 | HNF1A-AS1 | rs11065358 | T | C |  | NA | 0.63 | 0.63 | NA | 0.64 | 0.62 |
| 12 | 121388559 | HNF1A-AS1 | rs1696359 | T | C |  | NA | 0.65 | 0.66 | NA | 0.67 | 0.64 |
| 12 | 121388962 | HNF1A-AS1 | rs2650000 | A | C |  | 0.66 | 0.65 | 0.66 | NA | 0.67 | 0.65 |
| 12 | 121390078 | HNF1A-AS1 | rs2701194 | A | G |  | NA | 0.63 | 0.65 | NA | 0.66 | 0.63 |
| 12 | 121391671 | HNF1A-AS1 | rs2701175 | C | A |  | 0.63 | 0.61 | 0.63 | NA | 0.64 | 0.61 |
| 12 | 121392040 | HNF1A-AS1 | rs11065365 | G | A |  | NA | 0.55 | 0.56 | NA | 0.57 | 0.54 |
| 12 | 121392341 | HNF1A-AS1 | rs1732391 | C | T |  | 0.66 | 0.65 | 0.66 | NA | 0.67 | 0.65 |
| 12 | 121397875 | HNF1A-AS1 | rs6489786 | A | G |  | 0.66 | 0.65 | 0.66 | NA | 0.67 | 0.65 |
| 12 | 121398654 | HNF1A-AS1 | rs7954039 | A | C |  | 0.66 | 0.65 | 0.66 | NA | 0.67 | 0.65 |
| 12 | 121398657 | HNF1A-AS1 | rs7954331 | G | T |  | 0.66 | 0.65 | 0.66 | NA | 0.67 | 0.65 |

Table S1.1 (Continued)

| **Chr** | **Position¥** | **Gene** | **SNP** | **Allele** | |  | **Alt Allele Frequency** | | | | | |
| --- | --- | --- | --- | --- | --- | --- | --- | --- | --- | --- | --- | --- |
|  |  |  |  |  |  |  | **AS264** | **GARNET** | **GECCOCYTO** | **GECCOINIT** | **HIPFX** | **WHIMS** |
|  |  |  |  | **Ref** | **Alt** |  | **n=1,603** | **n=2,382** | **n=1,177** | **n=216** | **n=1,909** | **n=3,511** |
| 12 | 121403724 | HNF1A-AS1 | rs7953249 | G | A |  | 0.59 | 0.58 | 0.59 | NA | 0.60 | 0.58 |
| 12 | 121404155 | HNF1A-AS1 | rs7135337 | A | C |  | 0.58 | 0.57 | 0.58 | NA | 0.58 | 0.57 |
| 12 | 121404584 | HNF1A-AS1 | rs1920792 | C | T |  | 0.51 | 0.53 | 0.53 | NA | 0.51 | 0.53 |
| 12 | 121405126 | HNF1A-AS1 | rs2251468 | C | A |  | 0.66 | 0.65 | 0.66 | NA | 0.67 | 0.65 |
| 12 | 121405210 | HNF1A-AS1 | rs10774579 | C | T |  | 0.51 | 0.53 | 0.52 | NA | 0.51 | 0.53 |
| 12 | 121406293 | HNF1A-AS1 | rs2393792 | A | G |  | 0.50 | 0.53 | 0.52 | NA | 0.51 | 0.53 |
| 12 | 121406370 | HNF1A-AS1 | rs2243616 | G | T |  | 0.65 | 0.64 | 0.65 | NA | 0.66 | 0.63 |
| 12 | 121413027 | HNF1A-AS1 | rs148608463 | A | G |  | 0.66 | 0.65 | 0.66 | NA | 0.67 | 0.65 |
| 12 | 121413345 | HNF1A-AS1 | rs142632970 | G | A |  | 0.68 | 0.67 | 0.68 | NA | 0.69 | 0.67 |
| 12 | 121414915 | HNF1A-AS1 | rs2255531 | A | G |  | 0.65 | 0.64 | 0.65 | NA | 0.67 | 0.64 |
| 12 | 121415293 | HNF1A-AS1 | rs7139079 | G | A |  | 0.59 | 0.58 | 0.60 | NA | 0.60 | 0.58 |
| 12 | 121415390 | HNF1A-AS1 | rs2464190 | C | T |  | 0.58 | 0.55 | 0.59 | NA | 0.58 | 0.56 |
| 12 | 121416622 | HNF1A | rs1169289 | G | C |  | 0.57 | 0.54 | 0.58 | NA | 0.57 | 0.55 |
| 12 | 121416650 | HNF1A | rs1169288 | C | A |  | 0.68 | 0.67 | 0.68 | NA | 0.69 | 0.67 |
| 12 | 121416988 | HNF1A | rs2244608 | G | A |  | 0.68 | 0.67 | 0.68 | NA | 0.70 | 0.67 |
| 12 | 121419056 | HNF1A | rs1169286 | C | T |  | 0.58 | 0.56 | 0.57 | NA | 0.59 | 0.57 |
| 12 | 121419926 | HNF1A | rs1169284 | C | T |  | 0.68 | 0.68 | 0.69 | NA | 0.70 | 0.68 |
| 12 | 121420260 | HNF1A | rs7979473 | A | G |  | 0.60 | 0.61 | 0.62 | NA | 0.61 | 0.61 |
| 12 | 121420263 | HNF1A | rs7979478 | A | G |  | 0.60 | 0.60 | 0.61 | NA | 0.61 | 0.61 |
| 12 | 121420807 | HNF1A | rs1183910 | A | G |  | 0.69 | 0.68 | 0.69 | NA | 0.70 | 0.68 |
| 12 | 121423285 | HNF1A | rs11065384 | T | C |  | 0.68 | 0.68 | 0.69 | NA | 0.70 | 0.68 |
| 12 | 121423376 | HNF1A | rs7970695 | G | A |  | 0.62 | 0.61 | 0.62 | NA | 0.63 | 0.61 |
| 12 | 121423386 | HNF1A | rs11065385 | A | G |  | 0.69 | 0.68 | 0.69 | NA | 0.70 | 0.68 |
| 12 | 121423659 | HNF1A | rs9738226 | A | G |  | 0.62 | 0.61 | 0.62 | NA | 0.63 | 0.61 |
| 12 | 121423956 | HNF1A | rs2393791 | C | T |  | 0.62 | 0.61 | 0.62 | NA | 0.63 | 0.61 |
| 12 | 121424406 | HNF1A | rs2393776 | G | A |  | 0.62 | 0.61 | 0.62 | 0.60 | 0.63 | 0.61 |
| 12 | 121424490 | HNF1A | rs2243458 | T | C |  | 0.69 | 0.68 | 0.69 | 0.66 | 0.70 | 0.68 |
| 12 | 121424574 | HNF1A | rs2393775 | G | A |  | 0.62 | 0.61 | 0.62 | 0.60 | 0.63 | 0.61 |
| 12 | 121424861 | HNF1A | rs7310409 | A | G |  | 0.62 | 0.61 | 0.62 | 0.59 | 0.63 | 0.61 |
| 12 | 121426478 | HNF1A | rs1169292 | T | C |  | 0.69 | 0.68 | 0.69 | 0.67 | 0.70 | 0.68 |
| 12 | 121426594 | HNF1A | rs1169294 | A | G |  | 0.69 | 0.68 | 0.69 | 0.67 | 0.70 | 0.68 |
| 12 | 121431225 | HNF1A | rs1169300 | A | G |  | 0.70 | 0.69 | 0.71 | 0.69 | 0.72 | 0.70 |
| 12 | 121431300 | HNF1A | rs1169301 | T | C |  | 0.70 | 0.69 | 0.71 | 0.69 | 0.72 | 0.70 |
| 12 | 121432603 | HNF1A | rs2264782 | T | C |  | 0.65 | 0.63 | 0.66 | 0.63 | 0.66 | 0.65 |
| 12 | 121434833 | HNF1A | rs2259852 | A | G |  | 0.65 | 0.63 | 0.66 | 0.63 | 0.66 | 0.64 |
| 12 | 121435342 | HNF1A | rs2259820 | T | C |  | 0.70 | 0.69 | 0.71 | 0.69 | 0.72 | 0.70 |

Table S1.1 (Continued)

| **Chr** | **Position¥** | **Gene** | **SNP** | **Allele** | |  | **Alt Allele Frequency** | | | | | |
| --- | --- | --- | --- | --- | --- | --- | --- | --- | --- | --- | --- | --- |
|  |  |  |  |  |  |  | **AS264** | **GARNET** | **GECCOCYTO** | **GECCOINIT** | **HIPFX** | **WHIMS** |
|  |  |  |  | **Ref** | **Alt** |  | **n=1,603** | **n=2,382** | **n=1,177** | **n=216** | **n=1,909** | **n=3,511** |
| 12 | 121435427 | HNF1A | rs2464196 | A | G |  | 0.70 | 0.69 | 0.71 | 0.69 | 0.72 | 0.70 |
| 12 | 121435475 | HNF1A | rs2464195 | A | G |  | 0.65 | 0.63 | 0.66 | 0.63 | 0.66 | 0.64 |
| 12 | 121435587 | HNF1A | rs2259816 | T | G |  | 0.65 | 0.63 | 0.66 | 0.63 | 0.66 | 0.64 |
| 12 | 121438311 | HNF1A | rs1169306 | T | C |  | 0.65 | 0.63 | 0.66 | 0.63 | 0.66 | 0.64 |
| 12 | 121438844 | HNF1A | rs735396 | C | T |  | 0.65 | 0.63 | 0.66 | 0.63 | 0.66 | 0.64 |
| 12 | 121439192 | HNF1A | rs1169309 | T | G |  | 0.65 | 0.63 | 0.66 | 0.63 | 0.66 | 0.64 |
| 12 | 121439433 | HNF1A | rs1169310 | A | G |  | 0.65 | 0.63 | 0.66 | 0.63 | 0.66 | 0.64 |
| 12 | 121440731 | C12orf43 | rs1169311 | T | C |  | 0.65 | 0.63 | 0.66 | 0.63 | 0.66 | 0.64 |
| 12 | 121441461 | C12orf43 | rs1169312 | T | G |  | 0.65 | 0.63 | 0.66 | 0.63 | 0.66 | 0.64 |
| 12 | 121442670 | C12orf43 | rs1169313 | C | T |  | 0.65 | 0.63 | 0.65 | 0.63 | 0.66 | 0.64 |
| 12 | 121445808 | C12orf43 | rs2257962 | C | T |  | 0.65 | 0.63 | 0.65 | 0.63 | 0.66 | 0.64 |
| 12 | 121450384 | C12orf43 | rs2254971 | C | G |  | 0.63 | 0.61 | 0.64 | 0.60 | 0.63 | 0.62 |
| 12 | 121454622 | C12orf43 | rs1182933 | T | C |  | 0.70 | 0.69 | 0.71 | 0.69 | 0.72 | 0.70 |
| 19 | 45411941 | APOE | rs429358 | C | T |  | 0.85 | 0.87 | 0.87 | NA | 0.87 | 0.86 |
| 19 | 45418790 | APOC1 | rs5117 | C | T |  | NA | 0.79 | NA | NA | NA | 0.78 |
| 22 | 22190785 | MAPK1 | rs56398890 | A | T |  | 0.58 | 0.56 | 0.56 | 0.53 | 0.57 | 0.56 |
| 22 | 22202164 | MAPK1 | rs9607320 | T | C |  | 0.59 | 0.58 | 0.57 | 0.53 | 0.58 | 0.57 |

Alt, alternative; Chr, chromosome; GWA, genome-wide association; Ref, reference; SNP, single–nucleotide polymorphism.

¥ GRCh 37 coordinated.

Table S1.2. Allele frequencies of 68 SNPs from other GWA studies associated with pro-inflammatory phenotypes (total n = 10,798)

| **Chr** | **Positions¥** | **Gene** | **SNP** | **Allele** | |  | **Alt Allele Frequency** | | | | | |
| --- | --- | --- | --- | --- | --- | --- | --- | --- | --- | --- | --- | --- |
|  |  |  |  |  |  |  | **AS264** | **Garnet** | **Geccocyto** | **Geccoinit** | **Hipfx** | **Whims** |
|  |  |  |  | **Ref** | **Alt** |  | **n=1,603** | **n=2,382** | **n=1,177** | **n=216** | **n=1,909** | **n=3,511** |
| 1 | 27180088 | ZDHHC18 | rs75460349 | A | C |  | 0.02 | 0.03 | 0.02 | 0.02 | 0.03 | 0.02 |
| 1 | 40036847 | PABPC4 | rs2293476 | G | C |  | 0.21 | 0.22 | 0.24 | 0.25 | 0.23 | 0.23 |
| 1 | 40064961 | PABPC4/HEYL | rs12037222 | G | A |  | NA | 0.22 | 0.24 | 0.24 | 0.23 | 0.23 |
| 1 | 66085574 | LEPR | rs3790439 | A | T |  | 0.63 | 0.63 | 0.62 | 0.67 | 0.63 | 0.63 |
| 1 | 66102257 | LEPR | rs1805096 | A | G |  | 0.63 | 0.62 | 0.62 | 0.67 | 0.63 | 0.63 |
| 1 | 66161461 | LEPR | rs4420065 | T | C |  | 0.63 | 0.62 | 0.62 | 0.68 | 0.63 | 0.63 |
| 1 | 91530305 | ZNF644 | rs469772 | C | T |  | 0.20 | 0.20 | 0.19 | 0.19 | 0.18 | 0.19 |
| 1 | 154426264 | IL6R | rs4129267 | C | T |  | 0.41 | 0.40 | 0.41 | 0.40 | 0.40 | 0.41 |
| 1 | 154426970 | IL6R | rs2228145 | A | C |  | 0.41 | 0.40 | 0.41 | 0.40 | 0.41 | 0.41 |
| 1 | 159683438 | CRP | rs1800947 | C | G |  | 0.06 | 0.06 | NA | 0.06 | 0.06 | 0.05 |
| 1 | 159684186 | CRP | rs1417938 | T | A |  | 0.30 | 0.31 | 0.31 | 0.32 | 0.32 | 0.32 |
| 1 | 247601595 | NLRP3 | rs12239046 | C | T |  | 0.37 | 0.37 | 0.38 | 0.36 | 0.37 | 0.37 |
| 1 | 247612562 | NLRP3 | rs10925027 | C | T |  | 0.40 | 0.40 | NA | 0.42 | 0.40 | 0.39 |
| 2 | 629881 | TMEM18 | rs12995480 | C | T |  | 0.17 | 0.18 | 0.18 | 0.17 | 0.17 | 0.18 |
| 2 | 27730940 | GCKR | rs1260326 | C | T |  | 0.41 | 0.41 | 0.41 | NA | 0.41 | 0.40 |
| 2 | 88438050 | FABP1 | rs4246598 | C | A |  | 0.45 | 0.46 | 0.45 | 0.47 | 0.45 | 0.46 |
| 2 | 102744854 | IL1R1 | rs9284725 | A | C |  | 0.24 | 0.25 | 0.23 | 0.25 | 0.25 | 0.25 |
| 2 | 113838145 | IL1F10 | rs13409371 | G | A |  | 0.39 | 0.39 | 0.41 | 0.43 | 0.39 | 0.40 |
| 2 | 113841030 | IL1F10 | rs6734238 | A | G |  | 0.40 | 0.40 | 0.42 | 0.44 | 0.40 | 0.40 |
| 2 | 214033530 | IKZF2 | rs1441169 | G | A |  | 0.49 | 0.49 | 0.48 | 0.50 | 0.48 | 0.48 |
| 3 | 49891885 | TRAIP | rs2352975 | C | T |  | NA | 0.69 | NA | 0.70 | 0.69 | 0.69 |
| 3 | 170705693 | EIF5A2 | rs1514895 | A | G |  | 0.30 | 0.30 | 0.30 | 0.27 | 0.28 | 0.29 |
| 5 | 131839618 | IRF1 | rs4705952 | A | G |  | 0.25 | NA | 0.27 | 0.24 | 0.25 | 0.24 |
| 5 | 172191052 | DUSP1 | rs17658229 | T | C |  | 0.04 | NA | 0.04 | 0.03 | 0.04 | 0.04 |
| 6 | 32591588 | HLA-DQA1 | rs9271608 | A | G |  | 0.17 | NA | NA | NA | NA | 0.16 |
| 6 | 116314634 | FRK | rs12202641 | C | T |  | 0.41 | NA | 0.41 | 0.41 | 0.40 | 0.41 |
| 6 | 117114025 | GPRC6A | rs6901250 | G | A |  | 0.33 | 0.32 | 0.32 | 0.32 | 0.33 | 0.31 |
| 6 | 126851160 | CENPW | rs1490384 | T | C |  | 0.49 | 0.51 | 0.51 | 0.54 | 0.47 | 0.49 |
| 6 | 130371227 | L3MBTL3 | rs9385532 | C | T |  | 0.33 | 0.31 | 0.31 | 0.33 | 0.32 | 0.33 |
| 7 | 22759469 | IL6 | rs1880241 | G | A |  | 0.52 | 0.52 | 0.50 | 0.52 | 0.51 | 0.51 |
| 7 | 22766645 | IL6 | rs1800795 | G | C |  | 0.43 | 0.43 | 0.41 | 0.40 | 0.43 | 0.44 |
| 7 | 36084529 | EEPD1 | rs2710804 | T | C |  | 0.37 | 0.36 | 0.39 | 0.38 | 0.38 | 0.38 |
| 7 | 72971231 | BCL7B | rs13233571 | C | T |  | 0.11 | 0.13 | 0.11 | 0.13 | 0.12 | 0.12 |
| 8 | 9183358 | PPP1R3B | rs9987289 | G | A |  | 0.09 | 0.09 | 0.08 | 0.08 | 0.09 | 0.09 |
| 8 | 9183596 | PPP1R3B | rs4841132 | G | A |  | 0.09 | 0.09 | 0.08 | 0.08 | 0.09 | 0.09 |
| 8 | 117007850 | TRPS1 | rs2064009 | T | C |  | 0.42 | 0.43 | 0.40 | 0.39 | 0.41 | 0.41 |
| 8 | 126344208 | NSMCE2 | rs2891677 | C | T |  | 0.55 | 0.54 | 0.54 | 0.57 | 0.55 | 0.55 |

Table S1.2 (Continued)

| **Chr** | **Positions¥** | **Gene** | **SNP** | **Allele** | |  | **Alt Allele Frequency** | | | | | |
| --- | --- | --- | --- | --- | --- | --- | --- | --- | --- | --- | --- | --- |
|  |  |  |  |  |  |  | **AS264** | **Garnet** | **Geccocyto** | **Geccoinit** | **Hipfx** | **Whims** |
|  |  |  |  | **Ref** | **Alt** |  | **n=1,603** | **n=2,382** | **n=1,177** | **n=216** | **n=1,909** | **n=3,511** |
| 9 | 136142355 | ABO | rs643434 | G | A |  | 0.36 | 0.37 | 0.35 | 0.31 | 0.35 | 0.35 |
| 10 | 91007360 | LIPA | rs1051338 | T | G |  | 0.30 | 0.30 | 0.29 | 0.28 | 0.30 | 0.29 |
| 11 | 13357183 | ARNTL | rs10832027 | A | G |  | 0.32 | 0.32 | 0.31 | 0.36 | 0.32 | 0.32 |
| 11 | 47312892 | MADD | rs10838687 | T | G |  | 0.21 | 0.21 | 0.20 | 0.23 | 0.21 | 0.20 |
| 11 | 60021948 | MS4A4A | rs1582763 | G | A |  | 0.36 | 0.38 | 0.35 | 0.39 | 0.36 | 0.38 |
| 11 | 72496148 | STARD10 | rs7121935 | G | A |  | 0.34 | 0.37 | 0.38 | NA | 0.38 | 0.38 |
| 12 | 95855385 | METAP2 | rs11108056 | C | G |  | 0.44 | 0.43 | NA | 0.44 | 0.46 | 0.43 |
| 12 | 103483094 | ASCL1 | rs10745954 | G | A |  | 0.52 | 0.52 | 0.50 | 0.55 | 0.51 | 0.52 |
| 12 | 103537266 | C12orf42 | rs10778215 | A | T |  | 0.53 | 0.53 | 0.53 | 0.58 | 0.52 | 0.53 |
| 14 | 73011885 | RGS6 | rs2239222 | A | G |  | 0.36 | 0.36 | 0.34 | NA | 0.36 | 0.35 |
| 14 | 94838142 | SERPINA1/SERPINA2P | rs112635299 | G | T |  | 0.02 | 0.02 | 0.02 | 0.03 | 0.02 | 0.02 |
| 15 | 51745277 | DMXL2 | rs4774590 | G | A |  | 0.38 | 0.37 | 0.39 | 0.35 | 0.38 | 0.39 |
| 15 | 53728154 | WDR72 | rs1189402 | A | G |  | 0.37 | 0.37 | 0.39 | 0.38 | 0.36 | 0.38 |
| 15 | 60878030 | RORA | rs340005 | A | G |  | 0.38 | 0.37 | 0.37 | 0.38 | 0.39 | 0.38 |
| 15 | 60894965 | RORA | rs340029 | T | C |  | 0.39 | 0.37 | 0.37 | 0.38 | 0.39 | 0.37 |
| 16 | 51158710 | SALL1 | rs10521222 | C | T |  | NA | 0.04 | NA | 0.04 | 0.05 | NA |
| 16 | 53803574 | FTO | rs1558902 | T | A |  | 0.39 | 0.41 | 0.40 | 0.42 | 0.40 | 0.40 |
| 17 | 16097430 | NCOR1 | rs178810 | C | T |  | NA | 0.56 | 0.57 | 0.57 | 0.57 | 0.56 |
| 17 | 72699833 | CD300LF/RAB37 | rs10512597 | C | T |  | 0.19 | 0.19 | 0.18 | 0.16 | 0.18 | 0.19 |
| 18 | 12821593 | PTPN2 | rs2847281 | A | G |  | 0.39 | 0.39 | 0.38 | 0.39 | 0.40 | 0.40 |
| 18 | 12841176 | PTPN2 | rs2852151 | G | A |  | 0.39 | 0.39 | 0.38 | 0.39 | 0.40 | 0.40 |
| 18 | 55080437 | ONECUT2 | rs4092465 | A | G |  | 0.62 | 0.63 | NA | NA | 0.65 | 0.64 |
| 18 | 57897803 | MC4R | rs12960928 | T | C |  | 0.26 | 0.26 | 0.26 | 0.26 | 0.27 | 0.27 |
| 19 | 45395714 | TOMM40 | rs157581 | T | C |  | NA | 0.21 | 0.21 | NA | 0.20 | 0.21 |
| 19 | 45396144 | TOMM40 | rs11556505 | C | T |  | 0.13 | 0.13 | 0.12 | NA | 0.12 | 0.14 |
| 19 | 45396219 | TOMM40 | rs157582 | C | T |  | 0.23 | 0.21 | 0.20 | NA | 0.20 | 0.21 |
| 19 | 45422946 | APOC1 | rs4420638 | A | G |  | 0.17 | NA | NA | NA | NA | 0.17 |
| 20 | 43042364 | HNF4A | rs1800961 | C | T |  | 0.03 | 0.03 | NA | 0.04 | 0.03 | 0.03 |
| 20 | 62343956 | ZGPAT | rs2315008 | G | T |  | 0.31 | 0.32 | 0.32 | 0.30 | 0.31 | 0.33 |
| 21 | 40465534 | PSMG1 | rs2836878 | G | A |  | 0.27 | 0.27 | 0.25 | 0.28 | 0.27 | 0.27 |
| 22 | 39074737 | TOMM22 | rs6001193 | A | G |  | NA | 0.35 | 0.37 | 0.30 | 0.36 | 0.36 |

Alt, alternative; Chr, chromosome; GWA, genome-wide association; Ref, reference; SNP, single–nucleotide polymorphism.

¥ GRCh 37 coordinated.
