# Supplementary material for: Pro-inflammatory cytokine polymorphisms and interactions with dietary alcohol and estrogen, risk factors for invasive breast cancer using a post genome-wide analysis for gene–gene and gene–lifestyle interaction
Source: Sci Rep. 2021 Jan 13;11:1058. doi: 10.1038/s41598-020-80197-1 (PMC7807068; doi:10.1038/s41598-020-80197-1)
Supplement: Supplementary file 1 — Supplementary Information. [file 41598_2020_80197_MOESM1_ESM.zip › Table S2.2020July 13.docx]

Table S2.1. The second stage of random survival forest analysis: predictive value of variable in overall non-obese group (BMI < 30 kg/m^2^)

| **Variable*** | **Minimal Depth†** | **VIMP** | **C-index** | **Error**¶ | **Drop Error§** |
| --- | --- | --- | --- | --- | --- |
| *SALL1* rs10521222 | 1.5150 | 0.0954 | 0.6687 | 0.3313 | 0.1687 |
| Duration of oral contraceptive use | 1.6442 | 0.0781 | 0.7709 | 0.2291 | 0.1023 |
| *HLA-DQA1* rs9271608 | 2.8432 | 0.0295 | 0.8275 | 0.1725 | 0.0565 |
| Dietary alcohol | 2.8722 | 0.0083 | 0.8400 | 0.1600 | 0.0126 |
| *APOC1* rs4420638 | 2.9950 | 0.0167 | 0.8441 | 0.1559 | 0.0041 |
| Duration of E+P use | 3.1356 | 0.0192 | 0.8715 | 0.1285 | 0.0274 |
| *DUSP1* rs17658229 | 3.6426 | 0.0090 | 0.8750 | 0.1250 | 0.0035 |
| *TRAIP* rs2352975 | 3.7308 | 0.0099 | 0.8792 | 0.1208 | 0.0042 |
| Waist circumference | 3.7958 | 0.0018 | 0.8794 | 0.1206 | 0.0002 |
| Hip circumference | 3.8262 | 0.0021 | 0.8802 | 0.1198 | 0.0008 |
| *FRK* rs12202641 | 3.9904 | 0.0104 | 0.8833 | 0.1167 | 0.0031 |
| Depressive symptom | 4.0480 | 0.0014 | 0.8822 | 0.1178 | -0.0011 |
| Age at menopause | 4.0730 | 0.0025 | 0.8852 | 0.1148 | 0.0030 |
| Waist-to-hip ratio | 4.1340 | 0.0013 | 0.8820 | 0.1180 | -0.0033 |
| How many cigarettes per day | 4.2606 | 0.0002 | 0.8816 | 0.1184 | -0.0004 |
| Family income | 4.4624 | 0.0010 | 0.8789 | 0.1211 | -0.0026 |
| *METAP2* rs11108056 | 4.5104 | 0.0027 | 0.8802 | 0.1198 | 0.0012 |
| *SERPINA1/SERPINA2P* rs112635299 | 4.6160 | 0.0029 | 0.8794 | 0.1206 | -0.0008 |
| % calories from protein | 4.7380 | 0.0006 | 0.8772 | 0.1228 | -0.0022 |

BMI, body mass index; C-index, concordance index; E+P, exogenous estrogen + progestin; VIMP, variable of importance.

* Variables are ordered by minimal depth.

† Predictive value of variable was assessed via minimal depth in the nested random survival forest models. A lower value is likely to have a greater impact on prediction.

¶ The incremental error rate of each variable was estimated in the nested sequence of models starting with the top variable, followed by the model with the top 2 variables, then the model with the top 3 variables, and so on. For example, the 3^rd^ error rate was estimated from the 3^rd^ nested model (including the 1^st^, 2^nd^, and 3^rd^ variables).

**§** The drop error rate was estimated by the difference between the error rates from the nested models with a prior and the corresponding variable. For example, the drop error rate of the 2^nd^ variable was estimated by the difference between the error rates from the 1^nd^ and 2^rd^ nested models. The error rate for the null model is set at 0.5; thus, the drop error rate for the 1^st^ variable was obtained by subtracting the error rate (0.3313) from 0.5.

Table S2.2. The second stage of random survival forest analysis: predictive value of variable in overall obese group (BMI ≥ 30 kg/m^2^)

| **Variable*** | **Minimal Depth†** | **VIMP** | **C-index** | **Error¶** | **Drop Error§** |
| --- | --- | --- | --- | --- | --- |
| *HLA-DQA1* rs9271608 | 2.3924 | 0.0669 | 0.6786 | 0.3214 | 0.1786 |
| *SALL1* rs10521222 | 3.4086 | 0.0155 | 0.7024 | 0.2976 | 0.0238 |
| *DUSP1* rs17658229 | 3.6476 | 0.0135 | 0.7159 | 0.2841 | 0.0135 |
| Age at menopause | 4.1150 | -0.0006 | 0.7294 | 0.2706 | 0.0136 |
| Dietary alcohol | 4.3032 | 0.0016 | 0.7294 | 0.2706 | 0.0000 |
| % calories from protein | 4.3584 | 0.0009 | 0.7312 | 0.2688 | 0.0017 |
| *IRF1* rs4705952 | 4.3766 | 0.0063 | 0.7313 | 0.2687 | 0.0001 |
| How many cigarettes per day | 4.4386 | 0.0003 | 0.7185 | 0.2815 | -0.0127 |
| Depressive symptom | 4.4530 | 0.0003 | 0.7162 | 0.2838 | -0.0023 |
| Duration of oral contraceptive use | 4.4624 | 0.0066 | 0.7370 | 0.2630 | 0.0207 |
| *HNF1A-AS1* rs2243616 | 4.4916 | 0.0059 | 0.7492 | 0.2508 | 0.0122 |
| *HNF1A* rs1169289 | 4.5328 | 0.0074 | 0.7557 | 0.2443 | 0.0066 |
| *HNF1A-AS1* rs142632970 | 4.5596 | 0.0093 | 0.7499 | 0.2501 | -0.0059 |
| Hip circumference | 4.5942 | 0.0017 | 0.7475 | 0.2525 | -0.0024 |
| *HNF1A-AS1* rs2393792 | 4.5988 | 0.0060 | 0.7478 | 0.2522 | 0.0003 |
| *TRAIP* rs2352975 | 4.6018 | 0.0067 | 0.7523 | 0.2477 | 0.0046 |
| *TOMM40*rs157581 | 4.8662 | 0.0049 | 0.7551 | 0.2449 | 0.0028 |
| Family income | 4.9726 | -0.0005 | 0.7511 | 0.2489 | -0.0040 |
| Waist-to-hip ratio | 5.0534 | 0.0015 | 0.7491 | 0.2509 | -0.0021 |
| *HNF1A* rs1169286 | 5.1392 | 0.0048 | 0.7476 | 0.2524 | -0.0014 |
| *APOC1* rs4420638 | 5.1412 | 0.0068 | 0.7517 | 0.2483 | 0.0040 |
| Waist circumference | 5.2440 | 0.0002 | 0.7468 | 0.2532 | -0.0048 |
| *PTPN2* rs2852151 | 5.4400 | 0.0025 | 0.7453 | 0.2547 | -0.0015 |
| Duration of E+P use | 5.9998 | 0.0003 | 0.7443 | 0.2557 | -0.0011 |

BMI, body mass index; C-index, concordance index; E+P, exogenous estrogen + progestin; VIMP, variable of importance.

* Variables are ordered by minimal depth.

† Predictive value of variable was assessed via minimal depth in the nested random survival forest models. A lower value is likely to have a greater impact on prediction.

¶ The incremental error rate of each variable was estimated in the nested sequence of models starting with the top variable, followed by the model with the top 2 variables, then the model with the top 3 variables, and so on. For example, the 3^rd^ error rate was estimated from the 3^rd^ nested model (including the 1^st^, 2^nd^, and 3^rd^ variables).

**§** The drop error rate was estimated by the difference between the error rates from the nested models with a prior and the corresponding variable. For example, the drop error rate of the 2^nd^ variable was estimated by the difference between the error rates from the 1^nd^ and 2^rd^ nested models. The error rate for the null model is set at 0.5; thus, the drop error rate for the 1^st^ variable was obtained by subtracting the error rate (0.3214) from 0.5.

Table S2.3. The second stage of random survival forest analysis: predictive value of variable in non-viscerally obese group (waist-to-hip ratio ≤ 0.85)

| **Variable*** | **Minimal Depth†** | **VIMP** | **C-index** | **Error¶** | **Drop Error§** |
| --- | --- | --- | --- | --- | --- |
| Duration of oral contraceptive use | 1.9300 | 0.0655 | 0.6538 | 0.3462 | 0.1538 |
| *SALL1* rs10521222 | 2.1540 | 0.0528 | 0.7268 | 0.2732 | 0.0730 |
| *HLA-DQA1* rs9271608 | 2.6486 | 0.0451 | 0.8160 | 0.1840 | 0.0892 |
| Dietary alcohol | 3.3070 | 0.0055 | 0.8239 | 0.1761 | 0.0079 |
| Hip circumference | 3.5870 | 0.0063 | 0.8327 | 0.1673 | 0.0088 |
| *DUSP1* rs17658229 | 3.6798 | 0.0097 | 0.8353 | 0.1647 | 0.0027 |
| *APOC1* rs4420638 | 3.9084 | 0.0116 | 0.8388 | 0.1612 | 0.0035 |
| BMI | 3.9534 | 0.0047 | 0.8410 | 0.1590 | 0.0022 |
| How many cigarettes per day | 3.9884 | 0.0015 | 0.8412 | 0.1588 | 0.0002 |
| Duration of E+P use | 3.9972 | 0.0081 | 0.8570 | 0.1430 | 0.0157 |
| Waist circumference | 4.0442 | 0.0034 | 0.8532 | 0.1468 | -0.0038 |
| *TRAIP* rs2352975 | 4.1984 | 0.0080 | 0.8601 | 0.1399 | 0.0069 |
| Age at menopause | 4.3104 | 0.0007 | 0.8603 | 0.1397 | 0.0002 |
| Family income | 4.3710 | 0.0005 | 0.8581 | 0.1419 | -0.0023 |
| Depressive symptom | 4.4854 | -0.0001 | 0.8536 | 0.1464 | -0.0045 |
| *FRK* rs12202641 | 4.7768 | 0.0057 | 0.8549 | 0.1451 | 0.0013 |
| % calories from protein | 4.7942 | 0.0004 | 0.8537 | 0.1463 | -0.0012 |
| *IRF1* rs4705952 | 4.8214 | 0.0028 | 0.8552 | 0.1448 | 0.0015 |
| *PABPC4/HEYL* rs12037222 | 4.8222 | 0.0017 | 0.8538 | 0.1462 | -0.0014 |
| *TOMM40* rs157581 | 4.8346 | 0.0040 | 0.8545 | 0.1455 | 0.0007 |
| *METAP2* rs11108056 | 4.8968 | 0.0027 | 0.8554 | 0.1446 | 0.0008 |
| *TOMM40* rs157582 | 5.0638 | 0.0030 | 0.8536 | 0.1464 | -0.0017 |
| *CENPW* rs1490384 | 5.1156 | 0.0011 | 0.8541 | 0.1459 | 0.0005 |
| *ASCL1* rs10745954 | 5.1570 | 0.0008 | 0.8528 | 0.1472 | -0.0013 |
| *HNF4A* rs1800961 | 5.2150 | 0.0058 | 0.8549 | 0.1451 | 0.0021 |

BMI, body mass index; C-index, concordance index; E+P, exogenous estrogen + progestin; VIMP, variable of importance.

* Variables are ordered by minimal depth.

† Predictive value of variable was assessed via minimal depth in the nested random survival forest models. A lower value is likely to have a greater impact on prediction.

¶ The incremental error rate of each variable was estimated in the nested sequence of models starting with the top variable, followed by the model with the top 2 variables, then the model with the top 3 variables, and so on. For example, the 3^rd^ error rate was estimated from the 3^rd^ nested model (including the 1^st^, 2^nd^, and 3^rd^ variables).

**§** The drop error rate was estimated by the difference between the error rates from the nested models with a prior and the corresponding variable. For example, the drop error rate of the 2^nd^ variable was estimated by the difference between the error rates from the 1^nd^ and 2^rd^ nested models. The error rate for the null model is set at 0.5; thus, the drop error rate for the 1^st^ variable was obtained by subtracting the error rate (0.3462) from 0.5.

Table S2.4. The second stage of random survival forest analysis: predictive value of variable in viscerally obese group (waist-to-hip ratio > 0.85)

| **Variable*** | **Minimal Depth†** | **VIMP** | **C-index** | **Error¶** | **Drop Error§** |
| --- | --- | --- | --- | --- | --- |
| *SALL1* rs10521222 | 1.9816 | 0.0496 | 0.5749 | 0.4251 | 0.0749 |
| *HLA-DQA1* rs9271608 | 2.8716 | 0.0320 | 0.6649 | 0.3351 | 0.0900 |
| Age at menopause | 3.0838 | 0.0052 | 0.6908 | 0.3092 | 0.0259 |
| Depressive symptom | 3.1608 | 0.0079 | 0.6953 | 0.3047 | 0.0044 |
| BMI | 3.7136 | 0.0136 | 0.7383 | 0.2617 | 0.0430 |
| *TRAIP* rs2352975 | 3.8540 | 0.0131 | 0.7500 | 0.2500 | 0.0117 |
| Duration of oral contraceptive use | 3.9262 | 0.0109 | 0.7518 | 0.2482 | 0.0018 |
| Waist circumference | 4.0348 | 0.0062 | 0.7510 | 0.2490 | -0.0009 |
| *APOC1* rs4420638 | 4.1132 | 0.0147 | 0.7655 | 0.2345 | 0.0145 |
| % calories from protein | 4.1470 | 0.0008 | 0.7593 | 0.2407 | -0.0062 |
| *HNF1A* rs2243458 | 4.2426 | 0.0050 | 0.7706 | 0.2294 | 0.0113 |
| Hip circumference | 4.2778 | 0.0039 | 0.7679 | 0.2321 | -0.0027 |
| *DUSP1* rs17658229 | 4.3022 | 0.0079 | 0.7762 | 0.2238 | 0.0084 |
| *IKZF2* rs1441169 | 4.3748 | 0.0025 | 0.7696 | 0.2304 | -0.0066 |
| Duration of E+P use | 4.3870 | 0.0081 | 0.7695 | 0.2305 | -0.0001 |
| Dietary alcohol | 4.4846 | -0.0006 | 0.7694 | 0.2306 | 0.0000 |
| Family income | 4.4854 | -0.0008 | 0.7579 | 0.2421 | -0.0116 |
| How many cigarettes per day | 4.7442 | 0.0012 | 0.7566 | 0.2434 | -0.0013 |

BMI, body mass index; C-index, concordance index; E+P, exogenous estrogen + progestin; VIMP, variable of importance.

* Variables are ordered by minimal depth.

† Predictive value of variable was assessed via minimal depth in the nested random survival forest models. A lower value is likely to have a greater impact on prediction.

¶ The incremental error rate of each variable was estimated in the nested sequence of models starting with the top variable, followed by the model with the top 2 variables, then the model with the top 3 variables, and so on. For example, the 3^rd^ error rate was estimated from the 3^rd^ nested model (including the 1^st^, 2^nd^, and 3^rd^ variables).

**§** The drop error rate was estimated by the difference between the error rates from the nested models with a prior and the corresponding variable. For example, the drop error rate of the 2^nd^ variable was estimated by the difference between the error rates from the 1^nd^ and 2^rd^ nested models. The error rate for the null model is set at 0.5; thus, the drop error rate for the 1^st^ variable was obtained by subtracting the error rate (0.4251) from 0.5.

Table S2.5. The second stage of random survival forest analysis: predictive value of variable in non-viscerally obese group (waist circumference ≤ 88 cm)

| **Variable*** | **Minimal Depth†** | **VIMP** | **C-index** | **Error¶** | **Drop Error§** |
| --- | --- | --- | --- | --- | --- |
| Duration of oral contraceptive use | 1.7434 | 0.0798 | 0.6662 | 0.3338 | 0.1662 |
| *SALL1* rs10521222 | 1.7460 | 0.0885 | 0.7727 | 0.2273 | 0.1066 |
| *HLA-DQA1* rs9271608 | 2.6822 | 0.0328 | 0.8330 | 0.1670 | 0.0602 |
| *APOC1* rs4420638 | 3.3712 | 0.0134 | 0.8468 | 0.1532 | 0.0138 |
| Dietary alcohol | 3.4820 | 0.0042 | 0.8483 | 0.1517 | 0.0015 |
| Duration of E+P use | 3.5464 | 0.0136 | 0.8741 | 0.1259 | 0.0258 |
| Hip circumference | 3.5680 | 0.0035 | 0.8781 | 0.1219 | 0.0041 |
| *DUSP1* rs17658229 | 3.8936 | 0.0090 | 0.8791 | 0.1209 | 0.0010 |
| Depressive symptom | 3.9458 | 0.0016 | 0.8765 | 0.1235 | -0.0026 |
| *TRAIP* rs2352975 | 4.0168 | 0.0077 | 0.8797 | 0.1203 | 0.0032 |
| BMI | 4.1760 | 0.0024 | 0.8816 | 0.1184 | 0.0019 |
| How many cigarettes per day | 4.2500 | 0.0009 | 0.8802 | 0.1198 | -0.0014 |
| *FRK* rs12202641 | 4.3040 | 0.0088 | 0.8830 | 0.1170 | 0.0028 |
| *IRF1* rs4705952 | 4.5980 | 0.0029 | 0.8839 | 0.1161 | 0.0009 |
| Age at menopause | 4.6170 | 0.0022 | 0.8839 | 0.1161 | 0.0000 |
| Family income | 4.6730 | 0.0001 | 0.8822 | 0.1178 | -0.0017 |
| % calories from protein | 4.6828 | -0.0002 | 0.8800 | 0.1200 | -0.0023 |
| Waist-to-hip ratio | 4.7026 | 0.0001 | 0.8782 | 0.1218 | -0.0018 |
| *METAP2* rs11108056 | 4.8332 | 0.0026 | 0.8795 | 0.1205 | 0.0014 |
| *TOMM22* rs6001193 | 4.9652 | 0.0013 | 0.8780 | 0.1220 | -0.0015 |
| *HNF4A* rs1800961 | 4.9742 | 0.0062 | 0.8781 | 0.1219 | 0.0001 |

BMI, body mass index; C-index, concordance index; E+P, exogenous estrogen + progestin; VIMP, variable of importance.

* Variables are ordered by minimal depth.

† Predictive value of variable was assessed via minimal depth in the nested random survival forest models. A lower value is likely to have a greater impact on prediction.

¶ The incremental error rate of each variable was estimated in the nested sequence of models starting with the top variable, followed by the model with the top 2 variables, then the model with the top 3 variables, and so on. For example, the 3^rd^ error rate was estimated from the 3^rd^ nested model (including the 1^st^, 2^nd^, and 3^rd^ variables).

**§** The drop error rate was estimated by the difference between the error rates from the nested models with a prior and the corresponding variable. For example, the drop error rate of the 2^nd^ variable was estimated by the difference between the error rates from the 1^nd^ and 2^rd^ nested models. The error rate for the null model is set at 0.5; thus, the drop error rate for the 1^st^ variable was obtained by subtracting the error rate (0.3338) from 0.5.

Table S2.6. The second stage of random survival forest analysis: predictive value of variable in viscerally obese group (waist circumference > 88 cm)

| **Variable*** | **Minimal Depth†** | **VIMP** | **C-index** | **Error¶** | **Drop Error§** |
| --- | --- | --- | --- | --- | --- |
| *HLA-DQA1* rs9271608 | 2.0014 | 0.0708 | 0.6662 | 0.3338 | 0.1662 |
| *SALL1* rs10521222 | 2.6920 | 0.0191 | 0.6873 | 0.3127 | 0.0211 |
| Age at menopause | 2.8462 | 0.0051 | 0.7046 | 0.2954 | 0.0173 |
| *DUSP1* rs17658229 | 2.9672 | 0.0164 | 0.7278 | 0.2722 | 0.0232 |
| Dietary alcohol | 3.2168 | 0.0051 | 0.7329 | 0.2671 | 0.0051 |
| Duration of oral contraceptive use | 3.3770 | 0.0158 | 0.7501 | 0.2499 | 0.0172 |
| BMI | 3.5354 | 0.0063 | 0.7605 | 0.2395 | 0.0104 |
| *TRAIP* rs2352975 | 3.6106 | 0.0124 | 0.7684 | 0.2316 | 0.0080 |
| Depressive symptom | 3.7806 | 0.0031 | 0.7673 | 0.2327 | -0.0012 |
| *IRF1* rs4705952 | 3.8524 | 0.0090 | 0.7701 | 0.2299 | 0.0028 |
| Duration of E+P use | 3.9210 | 0.0047 | 0.7750 | 0.2250 | 0.0050 |
| *TOMM40* rs157581 | 4.0956 | 0.0075 | 0.7809 | 0.2191 | 0.0059 |
| % calories from protein | 4.1116 | -0.0001 | 0.7747 | 0.2253 | -0.0063 |
| Waist-to-hip ratio | 4.1368 | 0.0015 | 0.7755 | 0.2245 | 0.0008 |
| How many cigarettes per day | 4.2130 | 0.0011 | 0.7685 | 0.2315 | -0.0070 |
| Hip circumference | 4.2352 | 0.0029 | 0.7686 | 0.2314 | 0.0001 |
| Family income | 4.2872 | -0.0004 | 0.7659 | 0.2341 | -0.0027 |

BMI, body mass index; C-index, concordance index; E+P, exogenous estrogen + progestin; VIMP, variable of importance.

* Variables are ordered by minimal depth.

† Predictive value of variable was assessed via minimal depth in the nested random survival forest models. A lower value is likely to have a greater impact on prediction.

¶ The incremental error rate of each variable was estimated in the nested sequence of models starting with the top variable, followed by the model with the top 2 variables, then the model with the top 3 variables, and so on. For example, the 3^rd^ error rate was estimated from the 3^rd^ nested model (including the 1^st^, 2^nd^, and 3^rd^ variables).

**§** The drop error rate was estimated by the difference between the error rates from the nested models with a prior and the corresponding variable. For example, the drop error rate of the 2^nd^ variable was estimated by the difference between the error rates from the 1^nd^ and 2^rd^ nested models. The error rate for the null model is set at 0.5; thus, the drop error rate for the 1^st^ variable was obtained by subtracting the error rate (0.3338) from 0.5.

Table S2.7. The second stage of random survival forest analysis: predictive value of variable in active group (MET ≥ 10.0)

| **Variable*** | **Minimal Depth†** | **VIMP** | **C-index** | **Error¶** | **Drop Error§** |
| --- | --- | --- | --- | --- | --- |
| *SALL1* rs10521222 | 1.8312 | 0.0590 | 0.6400 | 0.3600 | 0.1400 |
| Duration of oral contraceptive use | 1.8792 | 0.0665 | 0.7202 | 0.2798 | 0.0803 |
| Duration of E+P use | 2.9986 | 0.0201 | 0.7871 | 0.2129 | 0.0669 |
| *HLA-DQA1* rs9271608 | 3.0014 | 0.0349 | 0.8355 | 0.1645 | 0.0484 |
| Age at menopause | 3.4048 | 0.0032 | 0.8373 | 0.1627 | 0.0018 |
| *APOC1* rs4420638 | 3.8620 | 0.0108 | 0.8387 | 0.1613 | 0.0014 |
| BMI | 3.9490 | 0.0068 | 0.8544 | 0.1456 | 0.0157 |
| Hip circumference | 4.1014 | 0.0034 | 0.8522 | 0.1478 | -0.0023 |
| Waist circumference | 4.1262 | 0.0030 | 0.8498 | 0.1502 | -0.0024 |
| *TRAIP* rs2352975 | 4.1908 | 0.0105 | 0.8574 | 0.1426 | 0.0076 |
| Depressive symptom | 4.2184 | 0.0005 | 0.8561 | 0.1439 | -0.0013 |
| Dietary alcohol | 4.3028 | 0.0016 | 0.8554 | 0.1446 | -0.0007 |
| How many cigarettes per day | 4.3924 | 0.0013 | 0.8514 | 0.1486 | -0.0040 |
| *DUSP1* rs17658229 | 4.4292 | 0.0044 | 0.8491 | 0.1509 | -0.0023 |
| % calories from protein | 4.5430 | 0.0017 | 0.8485 | 0.1515 | -0.0006 |
| Waist-to-hip ratio | 4.6398 | -0.0001 | 0.8453 | 0.1547 | -0.0032 |
| *HNF4A* rs1800961 | 4.6500 | 0.0104 | 0.8450 | 0.1550 | -0.0003 |
| *IKZF2* rs1441169 | 4.9052 | 0.0006 | 0.8401 | 0.1599 | -0.0049 |
| Family income | 5.4464 | -0.0014 | 0.8402 | 0.1598 | 0.0001 |

BMI, body mass index; C-index, concordance index; E+P, exogenous estrogen + progestin; VIMP, variable of importance.

* Variables are ordered by minimal depth.

† Predictive value of variable was assessed via minimal depth in the nested random survival forest models. A lower value is likely to have a greater impact on prediction.

¶ The incremental error rate of each variable was estimated in the nested sequence of models starting with the top variable, followed by the model with the top 2 variables, then the model with the top 3 variables, and so on. For example, the 3^rd^ error rate was estimated from the 3^rd^ nested model (including the 1^st^, 2^nd^, and 3^rd^ variables).

**§** The drop error rate was estimated by the difference between the error rates from the nested models with a prior and the corresponding variable. For example, the drop error rate of the 2^nd^ variable was estimated by the difference between the error rates from the 1^nd^ and 2^rd^ nested models. The error rate for the null model is set at 0.5; thus, the drop error rate for the 1^st^ variable was obtained by subtracting the error rate (0.3600) from 0.5.

Table S2.8. The second stage of random survival forest analysis: predictive value of variable in inactive group (MET < 10.0)

| **Variable*** | **Minimal Depth†** | **VIMP** | **C-index** | **Error¶** | **Drop Error§** |
| --- | --- | --- | --- | --- | --- |
| Duration of oral contraceptive use | 2.1310 | 0.0468 | 0.6080 | 0.3920 | 0.1080 |
| *SALL1* rs10521222 | 2.1542 | 0.0476 | 0.6789 | 0.3211 | 0.0710 |
| Dietary alcohol | 3.0432 | 0.0096 | 0.6990 | 0.3010 | 0.0201 |
| Hip circumference | 3.3008 | 0.0087 | 0.7202 | 0.2798 | 0.0212 |
| *HLA-DQA1* rs9271608 | 3.4472 | 0.0277 | 0.7842 | 0.2158 | 0.0640 |
| Waist circumference | 3.4908 | 0.0110 | 0.7893 | 0.2107 | 0.0051 |
| BMI | 3.5068 | 0.0105 | 0.7895 | 0.2105 | 0.0002 |
| *DUSP1* rs17658229 | 3.8378 | 0.0131 | 0.7930 | 0.2070 | 0.0035 |
| *TRAIP* rs2352975 | 3.8536 | 0.0106 | 0.8038 | 0.1962 | 0.0108 |
| Waist-to-hip ratio | 3.9774 | 0.0043 | 0.8021 | 0.1979 | -0.0016 |
| Depressive symptom | 4.0262 | 0.0021 | 0.8008 | 0.1992 | -0.0014 |
| Family income | 4.0774 | 0.0016 | 0.8008 | 0.1992 | 0.0000 |
| Age at menopause | 4.3282 | 0.0045 | 0.8050 | 0.1950 | 0.0042 |
| How many cigarettes per day | 4.3526 | 0.0026 | 0.8043 | 0.1957 | -0.0007 |
| *APOC1* rs4420638 | 4.3936 | 0.0094 | 0.8083 | 0.1917 | 0.0040 |
| % calories from protein | 4.6442 | 0.0003 | 0.8064 | 0.1936 | -0.0019 |
| *IRF1* rs4705952 | 4.6606 | 0.0023 | 0.8034 | 0.1966 | -0.0030 |
| *BCL7B* rs13233571 | 4.8950 | 0.0012 | 0.8027 | 0.1973 | -0.0006 |
| Duration of E+P use | 4.9022 | 0.0022 | 0.8086 | 0.1914 | 0.0058 |
| *TMEM18* rs12995480 | 4.9252 | 0.0020 | 0.8080 | 0.1920 | -0.0006 |
| *PABPC4/HEYL* rs12037222 | 5.0148 | 0.0009 | 0.8074 | 0.1926 | -0.0005 |
| *FRK* rs12202641 | 5.0434 | 0.0044 | 0.8092 | 0.1908 | 0.0017 |
| *TOMM40* rs157581 | 5.0866 | 0.0036 | 0.8112 | 0.1888 | 0.0020 |
| *TOMM40* rs157582 | 5.2364 | 0.0032 | 0.8119 | 0.1881 | 0.0007 |

BMI, body mass index; C-index, concordance index; E+P, exogenous estrogen + progestin; VIMP, variable of importance.

* Variables are ordered by minimal depth.

† Predictive value of variable was assessed via minimal depth in the nested random survival forest models. A lower value is likely to have a greater impact on prediction.

¶ The incremental error rate of each variable was estimated in the nested sequence of models starting with the top variable, followed by the model with the top 2 variables, then the model with the top 3 variables, and so on. For example, the 3^rd^ error rate was estimated from the 3^rd^ nested model (including the 1^st^, 2^nd^, and 3^rd^ variables).

**§** The drop error rate was estimated by the difference between the error rates from the nested models with a prior and the corresponding variable. For example, the drop error rate of the 2^nd^ variable was estimated by the difference between the error rates from the 1^nd^ and 2^rd^ nested models. The error rate for the null model is set at 0.5; thus, the drop error rate for the 1^st^ variable was obtained by subtracting the error rate (0.3920) from 0.5.

Table S2.9. The second stage of random survival forest analysis: predictive value of variable in low-fat diet group (% calories from SFA < 9.0)

| **Variable*** | **Minimal Depth†** | **VIMP** | **C-index** | **Error¶** | **Drop Error§** |
| --- | --- | --- | --- | --- | --- |
| Duration of oral contraceptive use | 2.7116 | 0.0511 | 0.6574 | 0.3426 | 0.1574 |
| *SALL1* rs10521222 | 3.6910 | 0.0361 | 0.7253 | 0.2747 | 0.0679 |
| Dietary alcohol | 4.4534 | 0.0042 | 0.7365 | 0.2635 | 0.0111 |
| *HLA-DQA1* rs9271608 | 4.5846 | 0.0284 | 0.8052 | 0.1948 | 0.0687 |
| Age at menopause | 5.0860 | 0.0019 | 0.8029 | 0.1971 | -0.0023 |
| *SERPINA1/SERPINA2P* rs112635299 | 5.4164 | 0.0020 | 0.7985 | 0.2015 | -0.0044 |
| *DUSP1* rs17658229 | 5.4644 | 0.0155 | 0.8045 | 0.1955 | 0.0060 |
| Hip circumference | 5.4706 | 0.0054 | 0.8211 | 0.1789 | 0.0165 |
| BMI | 5.7774 | 0.0046 | 0.8231 | 0.1769 | 0.0021 |
| *CRP* rs1800947 | 5.7954 | 0.0034 | 0.8187 | 0.1813 | -0.0045 |
| *FRK* rs12202641 | 5.8416 | 0.0101 | 0.8249 | 0.1751 | 0.0062 |
| *TMEM18* rs12995480 | 5.9186 | 0.0024 | 0.8311 | 0.1689 | 0.0062 |
| Family income | 6.0472 | 0.0014 | 0.8322 | 0.1678 | 0.0011 |
| How many cigarettes per day | 6.0548 | -0.0004 | 0.8230 | 0.1770 | -0.0091 |
| Waist circumference | 6.1696 | 0.0024 | 0.8225 | 0.1775 | -0.0005 |
| *APOC1* rs4420638 | 6.1816 | 0.0078 | 0.8291 | 0.1709 | 0.0066 |
| % calories from protein | 6.1896 | 0.0008 | 0.8246 | 0.1754 | -0.0045 |
| *CENPW* rs1490384 | 6.2710 | 0.0033 | 0.8281 | 0.1719 | 0.0035 |
| Depressive symptom | 6.2762 | 0.0007 | 0.8263 | 0.1737 | -0.0018 |
| *NSMCE2* rs2891677 | 6.3330 | 0.0009 | 0.8247 | 0.1753 | -0.0016 |
| *PABPC4* rs2293476 | 6.3670 | 0.0042 | 0.8255 | 0.1745 | 0.0009 |
| *IL1F10* rs6734238 | 6.4732 | 0.0027 | 0.8239 | 0.1761 | -0.0017 |
| *IRF1* rs4705952 | 6.5646 | 0.0029 | 0.8223 | 0.1777 | -0.0016 |
| Waist-to-hip ratio | 6.6972 | -0.0010 | 0.8211 | 0.1789 | -0.0012 |
| *PABPC4/HEYL* rs12037222 | 6.7134 | 0.0035 | 0.8208 | 0.1792 | -0.0003 |
| Duration of E+P use | 6.7924 | 0.0014 | 0.8211 | 0.1789 | 0.0004 |
| *NLRP3* rs12239046 | 6.9790 | 0.0008 | 0.8227 | 0.1773 | 0.0016 |
| *BCL7B* rs13233571 | 7.1880 | -0.0003 | 0.8216 | 0.1784 | -0.0011 |
| *TOMM40* rs157581 | 7.2324 | 0.0013 | 0.8189 | 0.1811 | -0.0027 |
| *FABP1* rs4246598 | 7.8270 | 0.0011 | 0.8163 | 0.1837 | -0.0026 |
| *PSMG1* rs2836878 | 7.8390 | 0.0046 | 0.8209 | 0.1791 | 0.0047 |

BMI, body mass index; C-index, concordance index; E+P, exogenous estrogen + progestin; VIMP, variable of importance.

* Variables are ordered by minimal depth.

† Predictive value of variable was assessed via minimal depth in the nested random survival forest models. A lower value is likely to have a greater impact on prediction.

¶ The incremental error rate of each variable was estimated in the nested sequence of models starting with the top variable, followed by the model with the top 2 variables, then the model with the top 3 variables, and so on. For example, the 3^rd^ error rate was estimated from the 3^rd^ nested model (including the 1^st^, 2^nd^, and 3^rd^ variables).

**§** The drop error rate was estimated by the difference between the error rates from the nested models with a prior and the corresponding variable. For example, the drop error rate of the 2^nd^ variable was estimated by the difference between the error rates from the 1^nd^ and 2^rd^ nested models. The error rate for the null model is set at 0.5; thus, the drop error rate for the 1^st^ variable was obtained by subtracting the error rate (0.3426) from 0.5.

Table S2.10. The second stage of random survival forest analysis: predictive value of variable in high-fat diet group (% calories from SFA **≥** 9.0)

| **Variable*** | **Minimal Depth†** | **VIMP** | **C-index** | **Error¶** | **Drop Error§** |
| --- | --- | --- | --- | --- | --- |
| *SALL1* rs10521222 | 1.9076 | 0.0544 | 0.6122 | 0.3878 | 0.1122 |
| Duration of oral contraceptive use | 2.1410 | 0.0492 | 0.6840 | 0.3160 | 0.0718 |
| *TRAIP* rs2352975 | 3.0474 | 0.0239 | 0.7420 | 0.2580 | 0.0580 |
| *HLA-DQA1* rs9271608 | 3.1260 | 0.0318 | 0.7957 | 0.2043 | 0.0537 |
| Waist circumference | 3.3558 | 0.0086 | 0.8056 | 0.1944 | 0.0099 |
| Hip circumference | 3.4182 | 0.0076 | 0.8081 | 0.1919 | 0.0025 |
| BMI | 3.4212 | 0.0118 | 0.8096 | 0.1904 | 0.0015 |
| Duration of E+P use | 3.5472 | 0.0113 | 0.8288 | 0.1712 | 0.0192 |
| Dietary alcohol | 3.8016 | 0.0035 | 0.8302 | 0.1698 | 0.0014 |
| *APOC1* rs4420638 | 3.8234 | 0.0132 | 0.8357 | 0.1643 | 0.0055 |
| *DUSP1* rs17658229 | 4.0042 | 0.0066 | 0.8379 | 0.1621 | 0.0021 |
| How many cigarettes per day | 4.0082 | 0.0028 | 0.8357 | 0.1643 | -0.0022 |
| Age at menopause | 4.0266 | 0.0026 | 0.8391 | 0.1609 | 0.0034 |
| Depressive symptom | 4.0388 | 0.0019 | 0.8384 | 0.1616 | -0.0007 |
| Waist-to-hip ratio | 4.1390 | 0.0032 | 0.8357 | 0.1643 | -0.0027 |
| Family income | 4.4242 | 0.0006 | 0.8330 | 0.1670 | -0.0027 |
| *IRF1* rs4705952 | 4.4804 | 0.0036 | 0.8323 | 0.1677 | -0.0007 |
| % calories from protein | 4.5160 | 0.0003 | 0.8304 | 0.1696 | -0.0019 |
| *TOMM40* rs157581 | 4.5588 | 0.0070 | 0.8341 | 0.1659 | 0.0037 |
| *METAP2* rs11108056 | 4.7800 | 0.0021 | 0.8334 | 0.1666 | -0.0007 |
| *TOMM40* rs157582 | 4.9274 | 0.0029 | 0.8335 | 0.1665 | 0.0001 |
| *NCOR1* rs178810 | 5.0048 | 0.0014 | 0.8374 | 0.1626 | 0.0038 |
| *LIPA* rs1051338 | 5.1060 | 0.0014 | 0.8342 | 0.1658 | -0.0031 |
| *HNF4A* rs1800961 | 5.5230 | 0.0032 | 0.8352 | 0.1648 | 0.0009 |

BMI, body mass index; C-index, concordance index; E+P, exogenous estrogen + progestin; VIMP, variable of importance.

* Variables are ordered by minimal depth.

† Predictive value of variable was assessed via minimal depth in the nested random survival forest models. A lower value is likely to have a greater impact on prediction.

¶ The incremental error rate of each variable was estimated in the nested sequence of models starting with the top variable, followed by the model with the top 2 variables, then the model with the top 3 variables, and so on. For example, the 3^rd^ error rate was estimated from the 3^rd^ nested model (including the 1^st^, 2^nd^, and 3^rd^ variables).

**§** The drop error rate was estimated by the difference between the error rates from the nested models with a prior and the corresponding variable. For example, the drop error rate of the 2^nd^ variable was estimated by the difference between the error rates from the 1^nd^ and 2^rd^ nested models. The error rate for the null model is set at 0.5; thus, the drop error rate for the 1^st^ variable was obtained by subtracting the error rate (0.3878) from 0.5.
