# Supplementary material for: Pro-inflammatory cytokine polymorphisms and interactions with dietary alcohol and estrogen, risk factors for invasive breast cancer using a post genome-wide analysis for gene–gene and gene–lifestyle interaction
Source: Sci Rep. 2021 Jan 13;11:1058. doi: 10.1038/s41598-020-80197-1 (PMC7807068; doi:10.1038/s41598-020-80197-1)
Supplement: Supplementary file 1 — Supplementary Information. [file 41598_2020_80197_MOESM1_ESM.zip › Table S3.2020July13.docx]

Table S3.1. Overall analysis: results from multivariate regression predicting breast cancer risk

| **Variable** | **HR**† **(95% CI)** | ***p*** |
| --- | --- | --- |
| **SNP (Ref/Alt)** |  |  |
| ***TRAIP* rs2352975 (C/T)** | **1.32 (1.10 - 1.60)** | **0.004** |
| **Behavioral factor*** |  |  |
| **Duration of oral contraceptive** | **1.46 (1.22 - 1.75)** | **2.66e-05** |
| **Duration of E+P** | **1.54 (1.06 - 2.22)** | **0.022** |
| **BMI** | 1.20 (0.92 - 1.57) | 0.177 |

Alt, alternative allele; BMI, body mass index; CI, confidence interval; E + P, exogenous estrogen + progestin; HR, hazard ratio; Ref, reference allele; SNP, single nucleotide polymorphism. Numbers in bold face are statistically significant.

† Multivariate regression was adjusted by family income, waist and hip circumferences, depressive symptom, number of cigarettes per day, % calories from protein, dietary alcohol, and age at menopause.

* Behavioral factors were analyzed as binary variables via cutoff values, where the cutoff level and/or higher reflects greater risk for breast cancer on the basis of random survival forest analysis: oral contraceptive at 5.1 years, E + P at 10 years, and BMI at 30 kg/m^2^.

Table S3.2. Stratified analysis: results from multivariate regression predicting breast cancer risk

| **Variable** | **HR**† **(95% CI)** | ***p*** |
| --- | --- | --- |
| **< Overall non–obese group (BMI < 30 kg/m^2^) (n = 7,179) >** | | |
| **SNPs (Ref/Alt)** |  |  |
| ***SALL1* rs10521222 (C/T)** | **5.64 (3.16 - 10.06)** | **4.54e-09** |
| ***APOC1* rs4420638 (A/G)** | **1.44 (1.03 - 2.00)** | **0.033** |
| **Behavioral factors*** |  |  |
| **Duration of oral contraceptive** | **1.75 (1.40 - 2.20)** | **1.33e-06** |
| **Duration of E+P** | **1.93 (1.28 - 2.92)** | **0.002** |
| **Dietary alcohol** | **1.63 (1.20 - 2.21)** | **0.002** |
| **< Non–viscerally obese group, WHR ≤ 0.85 (n = 7,251) >** | | |
| **SNPs (Ref/Alt)** |  |  |
| ***DUSP1* rs17658229 (T/C)** | **2.46 (1.69 - 3.58)** | **2.59e-06** |
| ***HLA-DQA1* rs9271608 (A/G)** | **2.58 (1.93 - 3.44)** | **1.37e-10** |
| ***SALL1* rs10521222 (C/T)** | **3.49 (2.07 - 5.87)** | **2.76e-06** |
| ***APOC1* rs4420638 (A/G)** | 1.35 (1.00 - 1.83) | 0.053 |
| **Behavioral factors*** |  |  |
| **Duration of oral contraceptive** | **1.77 (1.43 - 2.19)** | **2.02e-07** |
| **Duration of E+P** | **1.54 (1.01 - 2.34)** | **0.044** |
| **Dietary alcohol** | **1.75 (1.24 - 2.47)** | **0.001** |
| **Hip circumference** | 1.15 (0.86 - 1.54) | 0.360 |
| **< Non–viscerally obese group, WST ≤ 88 cm (n = 6,024) >** | | |
| **SNPs (Ref/Alt)** |  |  |
| ***SALL1* rs10521222 (C/T)** | **6.72 (3.32 - 13.60)** | **1.21e-07** |
| ***APOC1* rs4420638 (A/G)** | **1.51 (1.05 - 2.17)** | **0.028** |
| **Behavioral factors*** |  |  |
| **Duration of oral contraceptive** | **1.86 (1.46 - 2.39)** | **7.63e-07** |
| **Duration of E+P** | 1.44 (0.89 - 2.35) | 0.141 |
| **< Active group, MET ≥ 10.0 (n = 4,221) >** | | |
| **SNPs (Ref/Alt)** |  |  |
| ***HLA-DQA1* rs9271608 (A/G)** | **2.34 (1.64 - 3.34)** | **3.10e-06** |
| ***SALL1* rs10521222 (C/T)** | **5.33 (2.62 - 10.84)** | **3.93e-06** |
| **Behavioral factors*** |  |  |
| **Duration of oral contraceptive** | **1.41 (1.07 - 1.87)** | **0.016** |
| **Duration of E+P** | **2.29 (1.46 - 3.57)** | **0.0003** |
| **< High-fat diet group, % cal. from SFA ≥ 9.0 (n = 7,873) >** | | |
| **SNPs (Ref/Alt)** |  |  |
| ***TRAIP* rs2352975 (C/T)** | **1.47 (1.17 - 1.83)** | **0.001** |
| ***SALL1* rs10521222 (C/T)** | **4.95 (2.71 - 9.05)** | **2.07e-07** |
| **Behavioral factors*** |  |  |
| **Duration of oral contraceptive*** | **1.32 (1.08 - 1.63)** | **0.007** |
| **Duration of E+P*** | **1.68 (1.11 - 2.55)** | **0.015** |
| **BMI*** | 0.86 (0.63 - 1.16) | 0.312 |

Alt, alternative allele; BMI, body mass index; CI, confidence interval; E+P, exogenous estrogen + progestin; HR, hazard ratio; MET, metabolic equivalent; Ref, reference allele; SFA, saturated fatty acids; SNP, single nucleotide polymorphism; WHR, waist-to-hip ratio; WST, waist circumference;. Numbers in bold face are statistically significant.

† Multivariate regression was adjusted by family income, BMI, waist and hip circumferences, depressive symptom, number of cigarettes per day, % calories from protein, dietary alcohol, age at menopause, duration of oral contraceptive use, and E+P use; variables tested for risk factors and joint effect were not included as covariates in the multivariate regression.

* Behavioral factors were analyzed as binary variables via cutoff values, where the cutoff level and/or higher reflects greater risk for breast cancer on the basis of random survival forest analysis: oral contraceptive at 5.1 years and E + P at 10 years (all subgroups); dietary alcohol at 18 g/day (BMI subgroup) and 22 g/day (WHR subgroup); hip circumference at 103 cm (WHR subgroup); and BMI at 29 kg/m^2^ (SFA subgroup).
