# Supplementary material for: Pro-inflammatory cytokine polymorphisms and interactions with dietary alcohol and estrogen, risk factors for invasive breast cancer using a post genome-wide analysis for gene–gene and gene–lifestyle interaction
Source: Sci Rep. 2021 Jan 13;11:1058. doi: 10.1038/s41598-020-80197-1 (PMC7807068; doi:10.1038/s41598-020-80197-1)
Supplement: Supplementary file 1 — Supplementary Information. [file 41598_2020_80197_MOESM1_ESM.zip › Table S4.2020.July13.docx]

Table S4.1. Overall analysis: joint effect of opposed estrogen intake with combined risk genotypes and behavioral factors on breast cancer risk

|  | **Total** | |  |  | **E+P < 10 years** | |  |  | | **E+P ≥ 10 years** | |
| --- | --- | --- | --- | --- | --- | --- | --- | --- | --- | --- | --- |
| **n** | **HR**† **(95% CI)** | ***p**** |  | **n** | **HR**† **(95% CI)** | ***p**** |  | **n** | **HR**† **(95% CI)** | | ***p**** |
| Risk genotypes (*TRAIP* rs2352975 CT+TT)£ | | | | | | | | | | | |
| 0 | reference |  |  | 3,523 | reference |  |  | 114 | 1.17 (0.48 - 2.85) | | 0.735 |
| 1 | **1.32 (1.10 - 1.60)** | **0.004** |  | 6,256 | **1.32 (1.09 - 1.60)** | **0.005** |  | 286 | **2.13 (1.40 - 3.25)** | | **0.0004** |
|  | | | | | | | | | | | |
| Behavioral factors (oral contraceptive use, BMI, and E + P)¶ | | | | | | | | | | | |
| 0 | reference |  |  | 4,786 | reference |  |  | 255 | **1.72 (1.03 - 2.87)** | | **0.039** |
| 1 | **1.73 (1.40 - 2.13)** | **2.30e-07** |  | 4,993 | **1.70 (1.37 - 2.11)** | **1.10e-06** |  | 145 | **2.39 (1.39 - 4.09)** | | **0.002** |
|  | | | | | | | | | | | |
| Risk genotypes combined with behavioral factors§ | | | | | | | | | | | |
| 0 | reference |  |  | 1,741 | reference |  |  | 78 | 1.48 (0.46 - 4.75) | | 0.515 |
| 1 | **1.74 (1.26 - 2.40)** | **0.0008** |  | 4,827 | **1.74 (1.26 - 2.40)** | **0.0008** |  | 213 | **2.60 (1.45 - 4.67)** | | **0.001** |
| 2 | **2.45 (1.76 - 3.41)** | **1.16e-07** |  | 3,211 | **2.38 (1.70 - 3.34)** | **4.02e-07** |  | 109 | **3.56 (1.90 - 6.67)** | | **7.58e-05** |
| *p* _trend_ | | **< 2e-16** |  |  |  |  |  |  |  | |  |

BMI, body mass index; CI, confidence interval; E+P, exogenous estrogen + progestin; HR, hazard ratio. Numbers in bold face are statistically significant.

† Multivariate regression for risk genotype analysis was adjusted by family income, BMI, waist and hip circumferences, depressive symptom, number of cigarettes per day, % calories from protein, dietary alcohol, age at menopause, duration of oral contraceptive use, and E+P use (in total analysis); for behavioral factor analysis, variables tested for joint effect were not included as covariates in the multivariate regression.

* *p* values were adjusted to correct for multiple testing via the Benjamini-Hochberg approach.

£ The number of risk genotypes was defined as follows: 0 (none) vs. 1 (1 risk allele).

¶ The number of behavioral factors was defined as follows: 0 (null risk behavior) vs. 1 (1 or more risk behaviors).

§ The combined number of risk genotypes and behavioral factors was based on risk genotypes defined as 0 (low risk) and 1 (high risk) and based on behavioral factors defined as 0 (low risk) and 1 (high risk). The ultimate number of risk genotypes combined with behavioral factors was defined as 0 (low risk for genotypes and behaviors), 1 (high risk for either genotypes or behaviors), and 2 (high risk for both genotypes and behaviors).

Table S4.2. Stratification analysis: joint effect of opposed estrogen intake with combined risk genotypes and behavioral factors on breast cancer risk

|  | **Total** | |  |  | **E+P < 10 years** | |  |  | | **E+P ≥ 10 years** | |
| --- | --- | --- | --- | --- | --- | --- | --- | --- | --- | --- | --- |
| **n** | **HR**† **(95% CI)** | ***p**** |  | **n** | **HR**† **(95% CI)** | ***p**** |  | **n** | **HR**† **(95% CI)** | | ***p**** |
| **< Overall non-obese group, BMI < 30 kg/m^2^ (n = 7,179) >** | | | | | | | | | | | |
| Risk genotypes (*SALL1* rs10521222 TT and *APOC1* rs4420638 GG)£ | | | | | | | | | | | |
| 0 | reference |  |  | 2,170 | reference |  |  | 111 | 0.84 (0.20 - 3.46) | | 0.809 |
| 1 | **2.38 (1.77 - 3.21)** | **1.21e-08** |  | 4,680 | **2.26 (1.66 - 3.07)** | **1.67e-07** |  | 218 | **4.60 (2.79 - 7.60)** | | **2.45e-09** |
|  | | | | | | | | | | | |
| Behavioral factors (oral contraceptive use, dietary alcohol intake, and E + P)¶ | | | | | | | | | | | |
| 0 | reference |  |  | 4,284 | reference |  |  | 214 | 1.71 (0.95 - 3.09) | | 0.074 |
| 1 | **1.80 (1.44 - 2.25)** | **2.73e-07** |  | 2,566 | **1.73 (1.37 - 2.19)** | **3.66e-06** |  | 115 | **3.46 (1.96 - 6.12)** | | **1.96e-05** |
|  | | | | | | | | | | | |
| Risk genotypes combined with behavioral factors§ | | | | | | | | | | | |
| 0 | reference |  |  | 1,354 | reference |  |  | 73 | 1.11 (0.15 - 8.34) | | 0.918 |
| 1 | **3.17 (1.94 - 5.16)** | **3.59e-06** |  | 3,746 | **3.21 (1.97 - 5.23)** | **2.74e-06** |  | 179 | **5.13 (2.47 - 10.68)** | | **1.18e-05** |
| 2 | **5.51 (3.36 - 9.03)** | **1.30e-11** |  | 1,750 | **5.16 (3.13 - 8.51)** | **1.23e-10** |  | 77 | **12.35 (5.93 - 25.72)** | | **1.84e-11** |
| *p* _trend_ | | **1e-15** |  |  |  |  |  |  |  | |  |
|  |  |  |  |  |  |  |  |  |  | |  |
| **< Non visceral-obese group, WHR ≤ 0.85 (n = 7,251) >** | | | | | | | | | | | |
| Risk genotypes (*DUSP1* rs17658229 CC, *HLA-DQA1* rs9271608 GG, *SALL1* rs10521222 TT, and *APOC1* rs4420638 GG)£ | | | | | | | | | | | |
| 0 | reference |  |  | 4,142 | reference |  |  | 180 | 1.53 (0.74 - 3.16) | | 0.250 |
| 1 | **2.75 (2.21 - 3.41)** | **< 2e-16** |  | 2,785 | **2.76 (2.20 - 3.45)** | **< 2e-16** |  | 144 | **3.92 (2.32 - 6.62)** | | **3.49e-07** |
|  | | | | | | | | | | | |
| Behavioral factors (oral contraceptive use, hip circumference, dietary alcohol intake, and E + P)¶ | | | | | | | | | | | |
| 0 | reference |  |  | 2,179 | reference |  |  | 122 | 1.15 (0.42 - 3.15) | | 0.792 |
| 1 | **1.63 (1.19 - 2.24)** | **0.002** |  | 3,097 | **1.64 (1.20 - 2.26)** | **0.002** |  | 154 | **3.37 (1.90 - 6.00)** | | **3.45e-05** |
| 2 | **2.68 (1.89 - 3.78)** | **2.43e-08** |  | 1,651 | **2.57 (1.80 - 3.68)** | **2.33e-07** |  | 48 | **3.46 (1.38 - 8.71)** | | **0.008** |
|  | | | | | | | | | | | |
| Risk genotypes combined with behavioral factors§ | | | | | | | | | | | |
| 0 | reference |  |  | 1,360 | reference |  |  | 74 | 1.08 (0.14 - 8.15) | | 0.938 |
| 1 | **3.26 (1.96 - 5.43)** | **5.68e-06** |  | 3,601 | **3.27 (1.96 - 5.46)** | **5.79e-06** |  | 154 | **5.13 (2.34 - 11.24)** | | **4.37e-05** |
| 2 | **7.05 (4.22 - 11.77)** | **8.29e-14** |  | 1,966 | **6.99 (4.17 - 11.70)** | **1.46e-13** |  | 96 | **10.66 (5.11 - 22.21)** | | **2.70e-10** |
| *p* _trend_ | | **< 2e-16** |  |  |  |  |  |  |  | |  |
|  |  |  |  |  |  |  |  |  |  | |  |
| **< Non visceral-obese group, WST ≤ 88 cm (n = 6,024) >** | | | | | | | | | | | |
| Risk genotypes (*SALL1* rs10521222 TT and *APOC1* rs4420638 GG)£ | | | | | | | | | | | |
| 0 | reference |  |  | 1,802 | reference |  |  | 101 | 0.93 (0.22 - 3.85) | | 0.919 |
| 1 | **2.52 (1.81 - 3.52)** | **5.51e-08** |  | 3,933 | **2.45 (1.74 - 3.46)** | **2.82e-07** |  | 188 | **3.69 (2.05 - 6.67)** | | **1.48e-05** |
|  | | | | | | | | | | | |
| Behavioral factors (oral contraceptive use and E + P)¶ | | | | | | | | | | | |
| 0 | reference |  |  | 4,115 | reference |  |  | 226 | 1.29 (0.70 - 2.39) | | 0.412 |
| 1 | **1.81 (1.42 - 2.31)** | **1.7e-06** |  | 1,620 | **1.85 (1.43 - 2.39)** | **2.26e-06** |  | 63 | **2.78 (1.28 - 6.05)** | | **0.010** |
|  | | | | | | | | | | | |
| Risk genotypes combined with behavioral factors§ | | | | | | | | | | | |
| 0 | reference |  |  | 1,290 | reference |  |  | 78 | 0.85 (0.11 - 6.34) | | 0.873 |
| 1 | **3.00 (1.86 - 4.83)** | **6.88e-06** |  | 3,337 | **3.05 (1.89 - 4.92)** | **5.05e-06** |  | 171 | **4.16 (1.98 - 8.76)** | | **0.0001** |
| 2 | **5.31 (3.24 - 8.71)** | **3.56e-11** |  | 1,108 | **5.32 (3.22 - 8.80)** | **6.83e-11** |  | 40 | **8.65 (3.36 - 22.25)** | | **7.62e-06** |
| *p* _trend_ | | **4e-15** |  |  |  |  |  |  |  | |  |

Table S4.2 (Continued)

|  | **Total** | |  |  | **E+P < 10 years** | |  |  | | **E+P ≥ 10 years** | |
| --- | --- | --- | --- | --- | --- | --- | --- | --- | --- | --- | --- |
| **n** | **HR**† **(95% CI)** | ***p**** |  | **n** | **HR**† **(95% CI)** | ***p**** |  | **n** | **HR**† **(95% CI)** | | ***p**** |
| **< Active group, MET ≥ 10.0 (n = 4,221) >** | | | | | | | | | | | |
| Risk genotypes (*HLA-DQA1* rs9271608 GG and *SALL1* rs10521222 TT)£ | | | | | | | | | | | |
| 0 | reference |  |  | 1,711 | reference |  |  | 86 | 0.93 (0.22 - 3.89) | | 0.924 |
| 1 | **2.94 (2.11 - 4.09)** | **1.52e-10** |  | 2,305 | **2.73 (1.95 - 3.84)** | **6.44e-09** |  | 119 | **7.12 (4.17 - 12.14)** | | **5.86e-13** |
|  | | | | | | | | | | | |
| Behavioral factors (oral contraceptive use and E + P)¶ | | | | | | | | | | | |
| 0 | reference |  |  | 2,733 | reference |  |  | 158 | **2.37 (1.40 - 4.002)** | | **0.001** |
| 1 | **1.56 (1.19 - 2.05)** | **0.001** |  | 1,283 | **1.40 (1.05 - 1.88)** | **0.024** |  | 47 | **2.93 (1.27 - 6.76)** | | **0.012** |
|  | | | | | | | | | | | |
| Risk genotypes combined with behavioral factors§ | | | | | | | | | | | |
| 0 | reference |  |  | 1,184 | reference |  |  | 64 | 0.67 (0.09 - 4.94) | | 0.695 |
| 1 | **2.20 (1.45 - 3.33)** | **0.0002** |  | 2,076 | **2.24 (1.48 - 3.40)** | **0.0002** |  | 116 | **6.09 (3.27 - 11.34)** | | **1.25e-08** |
| 2 | **4.38 (2.83 - 6.78)** | **3.37e-11** |  | 756 | **3.83 (2.43 - 6.03)** | **7.33e-09** |  | 25 | **9.09 (3.50 - 23.61)** | | **5.77e-06** |
| *p* _trend_ | | **7e-11** |  |  |  |  |  |  |  | |  |
|  | | | | | | | | | | | |
| **< High-fat diet group, % cal. from SFA ≥ 9.0 (n = 7,873) >** | | | | | | | | | | | |
| Risk genotypes (*TRAIP* rs2352975 CT+TT and *SALL1* rs10521222 TT)£ | | | | | | | | | | | |
| 0 | reference |  |  | 3,291 | reference |  |  | 132 | 0.79 (0.25 - 2.50) | | 0.692 |
| 1 | **1.90 (1.52 - 2.37)** | **1.33e-08** |  | 4,273 | **1.86 (1.48 - 2.33)** | **6.98e-08** |  | 177 | **3.47 (2.16 - 5.56)** | | **2.46e-07** |
|  | | | | | | | | | | | |
| Behavioral factors (oral contraceptive use, BMI, and E + P)¶ | | | | | | | | | | | |
| 0 | reference |  |  | 3,278 | reference |  |  | 180 | 1.84 (1.01 - 3.33) | | 0.046 |
| 1 | **1.47 (1.14 - 1.89)** | **0.003** |  | 4,286 | **1.41 (1.09 - 1.84)** | **0.010** |  | 129 | **2.03 (1.11 - 3.74)** | | **0.022** |
|  | | | | | | | | | | | |
| Risk genotypes combined with behavioral factors§ | | | | | | | | | | | |
| 0 | reference |  |  | 1,580 | reference |  |  | 91 | 0.64 (0.09 - 4.72) | | 0.662 |
| 1 | **2.81 (1.85 - 4.26)** | **1.27e-06** |  | 3,409 | **2.85 (1.88 - 4.33)** | **9.27e-07** |  | 130 | **5.94 (3.04 - 11.60)** | | **1.81e-07** |
| 2 | **3.89 (2.54 - 5.98)** | **4.99e-10** |  | 2,575 | **3.70 (2.40 - 5.70)** | **3.04e-09** |  | 88 | **5.35 (2.55 - 11.24)** | | **9.51e-06** |
| *p* _trend_ | | **< 2e-16** |  |  |  |  |  |  |  | |  |

BMI, body mass index; CI, confidence interval; E+P, exogenous estrogen + progestin; HR, hazard ratio; MET, metabolic equivalent; SFA, saturated fatty acids; WHR, waist-to-hip ratio; WST, waist circumference. Numbers in bold face are statistically significant.

† Multivariate regression for risk genotype analysis was adjusted by family income, BMI, waist and hip circumferences, depressive symptom, number of cigarettes per day, % calories from protein, dietary alcohol, age at menopause, duration of oral contraceptive use, and E+P use (in total analysis); for behavioral factor analysis, variables tested for stratification and joint effect were not included as covariates in the multivariate regression.

* *p* values were adjusted to correct for multiple testing via the Benjamini-Hochberg approach.

£ The number of risk genotypes was defined as follows: [BMI < 30; WST ≤ 88; MET ≥ 10] 0 (none/1 risk allele) vs. 1 (2 risk alleles); [SFA ≥ 9] 0 (none/1/2 risk alleles) vs. 1 (3 risk alleles); [WHR ≤ 0.85] 0 (none/1/2/3 risk alleles) vs. 1 (4 risk alleles).

¶ The number of behavioral factors was defined as follows: [all subgroups except WHR subgroup] 0 (null risk behavior) vs. 1 (1 or more risk behaviors); [WHR ≤ 0.85] 0 (null risk behavior) vs. 1 (1 risk behavior) vs. 2 (2 or more risk behaviors).

§ The combined number of risk genotypes and behavioral factors was based on risk genotypes defined as 0 (low risk) and 1 (high risk) and based on behavioral factors defined as 0 (low risk) and 1 (high risk). The ultimate number of risk genotypes combined with behavioral factors was defined as 0 (low risk for genotypes and behaviors), 1 (high risk for either genotypes or behaviors), and 2 (high risk for both genotypes and behaviors).
